# Phenotypic and transcriptional profiling in *Entamoeba histolytica* reveal costs to fitness and adaptive responses associated with metronidazole resistance

**DOI:** 10.3389/fmicb.2015.00354

**Published:** 2015-05-05

**Authors:** Gil M. Penuliar, Kumiko Nakada-Tsukui, Tomoyoshi Nozaki

**Affiliations:** ^1^Department of Parasitology, National Institute of Infectious DiseasesTokyo, Japan; ^2^Department of Parasitology, Gunma University Graduate School of MedicineMaebashi, Japan; ^3^Graduate School of Life and Environmental Sciences, University of TsukubaTsukuba, Japan

**Keywords:** *Entamoeba histolytica*, metronidazole, drug resistance, transcriptome

## Abstract

Antimicrobial chemotherapy is critical in the fight against infectious diseases caused by *Entamoeba histolytica*. Among the drugs available for the treatment of amebiasis, metronidazole (MTZ) is considered the drug of choice. Recently, *in vitro* studies have described MTZ resistance and the potential mechanisms involved. Costs to fitness and adaptive responses associated with resistance, however, have not been investigated. In this study we generated an HM-1 derived strain resistant to 12 μM MTZ (MTZR). We examined its phenotypic and transcriptional profile to determine the consequences and mRNA level changes associated with MTZ resistance. Our results indicated increased cell size and granularity, and decreased rates in cell division, adhesion, phagocytosis, cytopathogenicity, and glucose consumption. Transcriptome analysis revealed 142 differentially expressed genes in MTZR. In contrast to other MTZ resistant parasites, MTZR did not down-regulate pyruvate:ferredoxin oxidoreductase, but showed increased expression of genes for a hypothetical protein (HP1) and several iron-sulfur flavoproteins, and downregulation of genes for leucine-rich proteins. Fisher's exact test showed 24 significantly enriched GO terms in MTZR, and a 3-way comparison of modulated genes in MTZR against those of MTZR cultured without MTZ and HM-1 cultured with MTZ, showed that 88 genes were specific to MTZR. Overall, our findings suggested that MTZ resistance is associated with specific transcriptional changes and decreased parasite virulence.

## Introduction

Currently, 500 million cases of amebiasis are reported each year (World Health Organization Amoebiasis, 1997). Most of these cases are children from developing nations, who are particularly vulnerable to infectious diseases (Marie and Petri, 2014). Among the drugs available for the treatment of amebic dysentery and amebic liver abscess, metronidazole (MTZ) is one of the most widely used, and is often considered the drug of choice (Löfmark et al., 2010). Treatment with MTZ is usually very effective, i.e., no clinical isolates with high levels of resistance have been observed. However, reports of patients refractory to the drug are documented (Koutsaimanis et al., 1979; Dooley and O'Morain, 1988). In addition, *in vitro* studies on the induction of MTZ resistance in *Entamoeba histolytica* are described (Samarawickrema et al., 1997; Wassmann et al., 1999). Coincidentally, clinical and *in vitro* resistance to the drug in *Trichomonas vaginalis* (Müller et al., 1980; Wright et al., 2010), *Giardia lamblia* (Müller et al., 2007; Tejman-Yarden et al., 2011), *Blastocystis* spp. (Mirza et al., 2011a,b), *Neisseria gonorrhoeae* (Yoshikawa et al., 1974), and several anaerobic bacteria (Pumbwe et al., 2007; Peláez et al., 2008; Tanih et al., 2011) have also been reported since the drug was introduced in 1960 (Figure S1) (Durel et al., 1960). Based on these findings, it is reasonable to assume that clinical resistance to MTZ might soon be reported in *E. histolytica*.

Unlike aerobic cells, anaerobic organisms are sensitive to MTZ because their electron transport proteins have sufficient negative redox potential that can activate MTZ (Samuelson, 1999). After entering the cell, the drug is reduced to a cytotoxic nitro radical anion by electron donors like thioredoxin reductase and ferredoxin (Fdx), which in turn serve as electron acceptors for pyruvate:ferredoxin oxidoreductase (PFOR) (Samuelson, 1999; Leitsch et al., 2007). The nitro radical anion is reduced further to a nitrosoimidazole, and further reduction leads to the formation of hydroxylamine and eventually to an amine (West et al., 1982; Moreno and Docampo, 1985; Ludlum et al., 1988). In *E. histolytica*, the actual mechanism of action is not fully understood, but evidences from other organisms suggest inhibition of DNA synthesis, and damage to DNA, protein, and other cell components by oxidation and adduct formation (Ludlum et al., 1988; Sisson et al., 2000; Leitsch et al., 2007).

A few mechanisms have been proposed to explain how pathogens survive MTZ treatment. The process varies among organisms, but the general principle includes at least one of the following: altered reduction efficiency (Leiros et al., 2004), drug inactivation (Ralph and Clarke, 1978), reduced drug uptake (Lacey et al., 1993), active efflux (Pumbwe et al., 2007), and increased DNA damage repair (Land and Johnson, 1999). In *E. histolytica*, Samarawickrema et al. (1997) reported the increased expression of iron-containing superoxide dismutase (Fe-SOD) in a strain resistant to 10 μM MTZ, while Wassmann et al. (1999) showed that in addition to Fe-SOD upregulation, resistance to 40 μM MTZ was associated with increased peroxiredoxin (Prx) expression and decreased expression of Fdx. On the contrary, Tazreiter et al. (2008) showed no substantial increase in Fe-SOD expression and less than 3-fold upregulation of Fdx and Prx in cells exposed to 50 μM MTZ. In addition, all three studies did not find any significant changes in PFOR expression, indicating that its downregulation may not be mandatory for low to modest drug resistance in this parasite. Studies on MTZ resistance in *T. vaginalis* and *G. lamblia* also showed variable results regarding the role of PFOR (Quon et al., 1992; Rasoloson et al., 2002; Müller et al., 2008; Leitsch et al., 2011). Strains of *T. vaginalis* deficient in PFOR activity, for example, showed only low levels of anaerobic resistance to MTZ (Rasoloson et al., 2002). In addition, some clinical strains exhibited resistance only under aerobic conditions, and were completely sensitive to MTZ in the absence of oxygen (Rasoloson et al., 2002). These strains also showed no decrease in PFOR activity (Rasoloson et al., 2002). Other reports, however, have shown decreased Fdx levels in MTZ resistant trichomonads (Ralph et al., 1974; Yarlett et al., 1986) and anaerobic resistance that was correlated with decreased PFOR activity (Kulda et al., 1989). From these results, it is conceivable that different pathways are involved in drug activation, and that various mechanisms for MTZ resistance exist in protozoa.

Currently, most of the work done in *E. histolytica* focused either on pathways known to activate, i.e., chemically reduce, MTZ (Leitsch et al., 2007) or antioxidants that counteract oxidative stress, such as pyruvate:ferredoxin oxidoreductase, superoxide dismutase, and peroxiredoxin (Samarawickrema et al., 1997; Wassmann et al., 1999). Fitness costs and adaptive responses have not yet been examined. In this study we generated an *E. histolytica* HM-1-derived isogenic strain resistant to 12 μM MTZ (MTZR). By comparing its phenotype and transcriptional profile against the parental strain, our goal was to identify phenotypic and transcriptional changes related to or involved in MTZ resistance. We compared the transcriptome of a single MTZR line cultured with MTZ [MTZR (+)] against MTZR cultured without the drug [MTZR (−)], and HM-1 exposed to MTZ [HM-1 (+)] to determine the significance of the genes we identified. Finally, to verify their involvement in resistance, some genes were overexpressed by episomal transfection, followed by drug challenge.

## Materials and methods

### Chemicals and reagents

MTZ, ornidazole, emetine, chloroquine, paromomycin, L-cysteine, and N-(trans-Epoxysuccinyl)-L-leucine 4-guanidinobutylamide (E-64) were purchased from Sigma-Aldrich (St. Louis, MO, USA), while tinidazole was acquired from LTK Laboratories (St. Paul, MN, USA). DNAzol reagent, TRIzol reagent, PLUS reagent, Lipofectamine, and geneticin (G418) were secured from Invitrogen (Carlsbad, CA, USA). All other chemicals were obtained from Wako Pure Chemical Industries (Osaka, Japan) unless otherwise stated. Drugs were dissolved either in distilled water or dimethyl sulfoxide (DMSO) to a stock concentration of 100 mM and stored at −30°C.

### Cultivation and induction of MTZ resistance

*E. histolytica* strain HM-1:IMSS cl6 (HM-1) (Diamond et al., 1972) was cultured under axenic conditions in 6 mL BI-S-33 medium (BIS) at 35.5°C in 13 × 100 mm Pyrex screw cap culture tubes (Corning, Corning, NY) (Diamond et al., 1978). Induction of MTZ resistance was initiated when cells in mid-logarithmic phase of growth were exposed to 1 μM of the drug until late logarithmic phase. In this study, cultures with cell concentrations between 4 × 10^4^ and 2 × 10^5^/mL were considered to be in mid-logarithmic phase, and corresponds to cell densities after 1 and 2 days, respectively, after the cultures were initiated with at 2–5 × 10^4^ trophozoites per mL. The drug concentration was increased by 1 μM until it reached 4 μM. Cells were maintained with the same dose for 4 weeks before further increase in drug concentration was made. A strain growing at 12 μM MTZ [MTZR (+)] was obtained approximately after 28 weeks, and was used in all experiments. MTZR was also cultured without the drug [MTZR (−)] for 24 h up to 1 week when we tested for reversibility of resistance and when samples were prepared for microarray.

### Cultivation of chinese hamster ovary (CHO) cells

CHO cells, a gift from Dr. Kentaro Hanada, were cultured in Ham's F-12 medium (GIBCO, Invitrogen Co., Auckland, New Zealand) with 10% fetal calf serum (Medical Biological Laboratory International, Woburn, MA, USA) in 25 cm^2^ canted neck culture flasks (IWAKI, Tokyo, Japan) at 37°C with humidified air and 5% CO_2_.

### Growth kinetics

After trophozoite cultures in mid-log phase of HM-1 and MTZR were placed on ice for 5 min to detach cells from the glass surface, cells were collected by centrifugation 500 × g for 5 min at room temperature. After centrifugation, spent medium was discarded and the pellet was resuspended in 1–2 mL of BIS medium. The cell number was estimated on a haemocytometer. Approximately 6 × 10^4^ trophozoites were inoculated in 6 mL BIS medium with or without 12 μM MTZ, and the cultures were examined every 24 h for 5 days. Trypan blue exclusion assay was used to determine the number of viable cells in duplicate cultures (Penuliar et al., 2011).

### Half maximal inhibitory concentration (IC_50_) and cross-resistance

Trophozoites in mid-log phase of HM-1 and MTZR were harvested, resuspended in BIS medium as described above, and the concentration was adjusted to 1 × 10^5^ cells/mL. About 1 × 10^4^ cells in 100 μL BIS were seeded per well of a 96-well microtiter plate (Iwaki, Tokyo, Japan) and incubated at 35.5°C for 1 h under anaerobic conditions using Anaerocult A (Merck, Darmstadt, Germany). One hundred microliters of BIS containing 2-fold increases in concentration of the drugs listed in Table 1 was added, and the plates incubated for 24 and 48 h. The medium was removed and replaced with 200 μL 10% WST-1 reagent (Roche, Indianapolis, IN, USA) in phosphate-buffered saline (PBS) and the plates incubated for 20–40 min. Optical density at 450 nm was measured using a DTX 880 Multimode Detector (Beckman Coulter, Fullerton, CA, USA). The percentage of viable cells was calculated after subtraction of background absorbance as % viability = (absorbance of treated cells/absorbance of untreated cells) × 100%. IC_50_ values were calculated using the sigmoidal dose-response equation in GraphPad Prism 5.0 (GraphPad Software, La Jolla, CA, USA). All experiments were repeated three times with two replicates per experiment. To assay the hydrogen peroxide sensitivity of the transformants harboring one of the following plasmids: HP1-HA, ISF1-HA, ISF2-HA, ISF4-HA, and pEhEx-HA, about 1 × 10^4^ cells were seeded per well of a 96-well microtiter plate and incubated at 35.5°C for 12–16 h under anaerobic conditions. The cells were then exposed to 25–1600 μM of hydrogen peroxide for 4 h. Then the cell viability was measured by using WST-1 reagent as described above.

**Table 1:** **IC_50_ of drugs used to treat amebiasis and hydrogen peroxide in HM-1 and MTZR**.

**Compound**	**Description**	**HM-1**	**MTZR**	**RI^a^**
Chloroquine	Amebicide	1562.7 ± 0.3	3125.3 ± 0.4	2.0
Emetine	Amebicide	12.8 ± 0.3	50.3 ± 0.3	3.9
Metronidazole	Amebicide	6.5 ± 0.3	12.9 ± 0.3	2.0
Ornidazole	Amebicide	6.5 ± 0.3	12.3 ± 0.3	1.9
Paromomycin	Amebcide	12.6 ± 0.4	25.1 ± 0.3	2.0
Tinidazole	Amebicide	12.6 ± 0.2	24.9 ± 0.3	2.0
Hydrogen peroxide	Stress inducer	244.2 ± 0.4	488.1 ± 0.3	2.0

a *Resistance index values are determined by dividing the IC_50_ of drug in MTZR by that in HM-1*.

### CHO monolayer destruction assay

Rate of CHO monolayer destruction was measured as described previously with minor modifications (Penuliar et al., 2011). Briefly, CHO cells in logarithmic phase of growth were prepared as follows. After spent medium was discarded, CHO cells were incubated with trypsin-EDTA at 37°C for 5 min to detach cells. Cells were transferred to a 50 mL tube containing 5 mL of Ham's F-12 medium and cell density was estimated on a haemocytometer. Cells were collected by centrifugation at 500 × g for 10 min at room temperature. After the supernatant was discarded, the cell pellet was re-suspended in Ham's F-12 medium at 1 × 10^5^ CHO cells per mL. Approximately 100 μL of the CHO suspension was dispensed into each well on a 96-well plate and the plate was incubated at 37°C as described (Penuliar et al., 2011) for 24–48 h until CHO cells form a monolayer. The medium was removed and the plates washed twice with warm PBS. Approximately 1 × 10^4^ cells of HM-1 and MTZR were resuspended in 200 μL Opti-MEM medium (GIBCO, Invitrogen Co., Auckland, New Zealand) and added to each well. The plates were incubated under anaerobic conditions at 35.5°C for 15 min intervals up to 2 h. The plates were placed on ice for 15 min to release adhered trophozoites and washed twice with cold PBS. The percentage of CHO monolayer destroyed was determined relative to wells without the ameba using 10% WST-1.

### Substrate gel electrophoresis

Proteinase activity was detected by substrate gel electrophoresis as described previously (Hellberg et al., 2000). Briefly, 20 μg of cell lysate from HM-1 and MTZR strains were separated in a 12% [w/v] sodium dodecyl sulfate (SDS)-polyacrylamide gel copolymerized with 0.1% [w/v] gelatin. The gel was incubated in 2.5% [v/v] Triton X-100 for 1 h and then in 100 mM sodium acetate, pH 4.5, 1% [v/v] Triton X-100, and 20 mM dithiothreitol for 3 h at 37°C. Bands were visualized after staining with 0.5% [w/v] Coomassie Brilliant Blue R-250.

### Phagocytosis assay

Trophozoites of HM-1 and MTZR in mid-logarithmic phase of growth were harvested, resuspended in BIS medium as described above, and the concentration was adjusted to 1 × 10^6^ cells/mL. Approximately 1.0 × 10^6^ of HM-1 and MTZR trophozoites were seeded on each well of 12-well plates. Spent medium was removed and replaced with 500 μL warmed BIS with 1.5 × 10^7^ FluoSpheres carboxylate-modified (2.0 μm) Nile Red fluorescent beads (Invitrogen, Eugene, OR, USA). The plates were sealed and incubated at 35.5°C for up to 80 min. After incubation, 500 μL of PBS containing 2% galactose was added and the plates incubated on ice for at least 20 min. Formaldehyde was added to a final concentration of 4% and plates incubated on ice for 1 h. The cells were collected, washed three times with cold PBS and analyzed by flow cytometry as previously described (Nakada-Tsukui et al., 2005). Cells that ingested the beads were gated based on increased fluorescence compared to control non-phagocytic cells.

### Glucose consumption

Trophozoites of HM-1 and MTZR in mid-logarithmic phase of growth were harvested, resuspended in BIS medium as described above, and the concentration was adjusted to 1 × 10^7^ cells/mL. Approximately 1 × 10^7^ cells were seeded in 6-well dishes and incubated for 12 and 24 h. Spent medium was decanted and centrifuged at 500 × g for 5 min. Glucose consumption was determined from the clarified medium using a Glucose (GO) Assay kit (Sigma Aldrich, Saint Louis, MO, USA) according to manufacturer's protocol.

### Estimation of total protein amount in a single cell

Trophozoites of HM-1 and MTZR in mid-logarithmic phase of growth were harvested as described above. Cell pellet was re-suspended in PBS at 1 × 10^6^/mL. Approximately 500 μL of the cell suspension were dispensed to a 1.5 mL tube and the cells were collected by centrifugation at 500 × g for 3 min at 4°C. After the supernatant was carefully removed, the cell pellet was lysed with 50 μL of lysis buffer [50 mM Tris-HCl, pH 7.5, 150 mM NaCl, 1% Triton-X 100, complete mini EDTA-free protease inhibitor cocktail (Roche Molecular Biochemicals, Mannheim, Germany) and 200 mM E-64]. Protein amount of the lysate were quantified by *DC* protein assay kit (Bio-Rad Laboratories, Inc., Hercules, CA, USA).

### RNA isolation

HM-1 and MTZR were cultured with and without 12 μM MTZ in 25 cm^2^ tissue culture flasks. HM-1 cultured with the drug was incubated for 5 h [(HM-1 (+)], while MTZR without MTZ was maintained for 1 week [(MTZR (−)]. Except for HM-1 (+), the spent medium for all other cultures were removed and replaced with fresh BIS with and without the drug and the flasks incubated at 35.5°C for 5 h. Total RNA was extracted with TRIzol Reagent according to the manufacturer's instructions for cells grown in monolayers; cleaned with RNeasy kit (Qiagen, Hilden, Germany), and assessed for quality with the Experion automated electrophoresis system and Experion RNA StdSens analysis kit (Bio-Rad Laboratories, Inc., Hercules, CA, USA). RNA quantity was determined by measuring the absorbance at 260 nm with NanoDrop ND-1000 UV-Vis spectrophotometer (NanoDrop Technologies, Wilmington, DE, USA).

### Microarray hybridization

Samples from two independent RNA extractions were processed using Ambion MessageAmpTM Premier RNA Amplification Kit (Applied Biosystems, Foster City, CA, USA) according to manufacturer's instructions. Fragmented cRNA was then hybridized onto a probe array chip (Eh_Eia520620F) that was custom-made by Affymetrix (Santa Clara, CA, USA) (Husain et al., 2011; Penuliar et al., 2012) using kits and protocols specified in the Affymetrix GeneChip Expression Analysis Technical Manual (Affymetrix, Santa Clara, CA, USA). Following hybridization, arrays were washed, stained with streptavidin-phycoerythrin (Molecular Probes, Eugene, OR, USA) using Affymetrix GeneChip Fluidics Station 450, and scanned with an Affymetrix GeneChip Scanner 3000 at 570 nm. Each array image was visually screened to check for scratches, signal artifacts, and debris.

The microarray used in this study was made based on *E. histolytica* and *E. invadens* sequences stored at TIGR and Pathema databases (Loftus et al., 2005; http://amoebadb.org/amoeba/). It contained 9230 probe sets for *E. histolytica* and an additional 25 and 81 control probe sets for *Entamoeba* and Affymetrix, respectively (Eh_Eia520620F_Eh). Nomenclature for the IDs was based on whether the probe set is unique to either Pathema (e.g., EHI_123456) or TIGR (e.g., 12.m00345), or is found in both databases (e.g., 98.m00765_234567). Probe sets labeled with “_at” represent a single gene, while those labeled with “_s_at” represent probe sets that share all probes identically with at least two sequences. The “_s_at” probe sets represent highly similar transcripts, shorter forms of alternatively polyadenylated transcripts, or common regions in the 3′ ends of multiple alternative splice forms. Probe sets labeled with “_x_at,” on the other hand, represent probe sets where it was not possible to select either a unique probe set or a probe set with identical probes among multiple transcripts. These probe sets could cross-hybridize with other genes in an unpredictable manner (Affymetrix, Santa Clara, CA, USA).

### Data normalization, analysis, and deposition

Raw probe intensities were generated by the GeneChip Operating Software (GCOS) and GeneTitan Instrument from Affymetrix. Resulting expression values were analyzed by R/BioConductor and GeneSpring GX Ver.11.5 to identify differentially expressed genes. Reproducibility of the experiments was determined by Pearson's correlation coefficient and confirmed by principal component analysis (Pearson, 1901; Stigler, 1989). Only genes that were considered “present” by GCOS in both arrays were used in further analysis. Gene probe sets were considered differentially expressed between samples if they had at least a 3-fold change and a *P*-value < 0.05, calculated using Welch's *t*-test (Welch, 1947), after multiple test correction by the Benjamini–Hochberg method (Benjamini and Hochberg, 1995). A *post-hoc* test using Tukey's Honestly Significant Difference test was conducted to determine significant differences between samples (Tukey, 1949). Probe sets ending with “_x_at” were removed together with redundant genes identified by sequence alignment using ClustalW2.48 Finally, RefSeq's annotated pseudogenes were omitted from the final list (ClustalW2; http://www.ebi.ac.uk/Tools/msa/clustalw2/).

Nucleotide sequences of differentially expressed genes were searched against GenBank (http://www.ncbi.nlm.nih.gov/genbank/) and Amoeba DB (http://amoebadb.org/amoeba/) by using Basic Local Alignment Search Tool (BLAST; http://blast.ncbi.nlm.nih.gov/Blast.cgi). Matches against hypothetical proteins (HP) with *E*-value < e^−50^ and maximal scoring pair alignment that covered at least 40% were considered as putative orthologs (McLysaght et al., 2003). Hierarchical clustering of modulated genes in MTZR, MTZR (−), and HM-1 (+) was performed with Cluster 3.0 (http://bonsai.hgc.jp/~mdehoon/software/cluster/software.htm) using average linkage, while dendrograms and heatmaps were generated with Java TreeView version 1.1.6r2. (http://jtreeview.sourceforge.net) Gene Ontology (GO) annotations were mined from the Gene Ontology Annotation Database (http://www.ebi.ac.uk), and Amoeba DB. GO term enrichment was computed using Fisher's exact test (Agresti, 1992), against a background consisting of all GO IDs for genes symbols beginning with EHI, which were downloaded from the QuickGO database of European Molecular Biology Laboratory - European Bioinformatics Institute (EMBL-EBI) (Taxon ID: 5759) (http://www.ebi.ac.uk/QuickGO/). Genomic location of each gene was obtained from Pathema-Entamoeba (http://pathema.jcvi.org) while transmembrane domain prediction was performed using the following online servers: HMMTOP (http://www.enzim.hu), SOSUI (http://bp.nuap.nagoya-u.ac.jp), TMHMM (http://www.cbs.dtu.dk), TopPred (http://mobyle.pasteur.fr), TMpred (http://www.ch.embnet.org), and Phobius (http://phobius.sbc.su.se). Signal peptide sequences were predicted using SignalP (http://www.cbs.dtu.dk), SOSUIsignal (http://bp.nuap.nagoya-u.ac.jp), and Phobius. Putative transmembrane domains and signal peptides were considered present only if positive hits were found in all programs used. Venn diagrams were constructed using Venn Diagram Generator (http://www.pangloss.com/seidel/Protocols/venn.cgi).

The microarray data reported in this paper has been deposited in the Gene Expression Omnibus (GEO) database (http://www.ncbi.nlm.nih.gov/geo/) with accession number GSE35990.

### Generation of transgenic ameba overexpressing HP1 and ISFs

Full-length gene sequences were amplified from MTZR cDNA with sense- and anti-sense primers containing appropriate restriction sites (Table S2). PCR primers were designed as follows: sense primers contained 26~30 nucleotides corresponding to a region of each gene encoding the amino-terminal portion of each target proteins, fused at the 5′ end with three additional nucleotides and SmaI restriction enzyme recognition site (tccCCCGGG); reverse primers contained 22~33 nucleotides corresponding to a region of each gene encoding the carboxyl-terminal portion of each target proteins, fused at the 5′ end with three additional nucleotides and XhoI restriction enzyme recognition site (ccgCTCGAG). PCR was performed with the cDNA as a template using a DNA Engine Peltier Thermal Cycler (Bio-Rad, Hercules, CA, USA). The PCR cycling conditions consisted of an initial step of denaturation at 94°C for 1 min, followed by 30 cycles of denaturation at 98°C for 10 s, annealing at 55°C for 30 s, and extension at 72°C for 60 s with Phusion DNA polymerase (New England BioLabs, Tokyo, Japan). Resultant PCR fragments were precipitated with ethanol, digested with appropriate restriction enzymes, and then purified from an exised piece of agarose gel by using GENECLEAN kit (Funakoshi, Tokyo, Japan). After purification, the gene was inserted into an expression plasmid, pEhEx, as previously described (Penuliar et al., 2012). The plasmid was introduced into *E. histolytica* G3 strain by lipofection, with minor modifications as previously described (Penuliar et al., 2012). Briefly, *E. histolytica* G3 trophozoites in the mid- logarithmic growth phase were harvested as described above, and re-suspended with Opti-MEM medium supplemented with 5 mg/mL L-cysteine and 1 mg/mL ascorbic acid at 1 × 10^5^ cells/mL. Approximately 5 mL of the suspension was dispensed into each well on a 12-well plate and the plate was incubated under anaerobic condition at 35.5°C for 30 min. Following incubation, 4.5 mL medium from each well was removed and 500 μL liposome–plasmid–mixture (5 μg plasmid, 10 μL PLUS reagent, 20 μL Lipofectamine in Opti-MEM medium) was added. After 5 h of transfection, cells were harvested by placing the plate on ice for 15 min, then added to culture tubes with 5.5 mL cold BIS, and incubated at 35.5°C for 24 h. Transformants were initially selected in the presence of 1 μg/mL of G418, and drug concentration was gradually increased to 10 μg/mL during the following 6 weeks before the transformants were analyzed.

### Reverse transcriptase (RT)-PCR and quantitative real-time (qRT)-PCR

Total RNA from semi-confluent cultures, about 2 × 10^5^ cells/mL, of HM-1, MTZR, and transformants harboring one of the following plasmids: pEhEx-HA (mock plasmid), HP1-HA, ISF1-HA, ISF2-HA, and ISF4-HA, cultured in 25 cm^2^ flasks, was extracted and quantified as described previously (Penuliar et al., 2012). cDNA was reverse transcribed from 5 μg RNA with the SuperScript III First-Strand Synthesis System, and RT-PCR was performed with the resulting cDNA as template and primers listed in Table S2 on a DNA Engine Peltier Thermal Cycle (Bio-Rad Laboratories, Inc., Hercules, CA, USA). The PCR parameters used were: an initial denaturation step at 95°C for 2 min followed by 30 cycles of denaturation at 94°C for 15 s, annealing at 55°C for 30 s, and extension at 72°C for 30 or 60 s. The products were resolved on 1.5% [w/v] agarose gel with 0.5 μg/mL ethidium bromide, visualized, and photographed under ultraviolet illumination.

The Fast SYBR Green Master Mix (Applied Biosystems, Foster City, CA, USA) was used for qRT-PCR in accordance with the manufacturer's instructions. The list of primers for genes whose expression was quantified by qRT-PCR can be found in Table S1, and includes a housekeeping gene, RNA polymerase II gene (rpoII), as control. Each PCR reaction contained 5 μL (1:50 dilution) of cDNA and 15 μL primer mix, composed of 10 μL 2X Fast SYBR Green Master Mix, sense and antisense primers, and nuclease-free water, to bring the volume to 20 μL. qRT–PCR was performed using StepOne Plus Real-Time PCR System (Applied Biosystems, Foster City, CA, USA) with the following cycling conditions: enzyme activation at 95°C for 20 s, followed by 40 cycles of denaturation at 95°C for 3 s and annealing/extension at 60°C for 30 s. All test samples were run in triplicate including an RT-negative control for each sample set along with a blank control consisting of nuclease-free water in place of cDNA. Quantification for each target gene was determined by the ΔΔCt method with rpoII as reference gene (Livak and Schmittgen, 2001).

### Statistical analysis

Correlation coefficients were calculated using the Student's *t*-test function of Microsoft Excel statistical package (Microsoft Corp., Redmond, WA, USA). Probability levels (*P*) < 0.05 were considered significant.

## Results

### Generation and phenotypic characterization of MTZR

Exposure of HM-1 to MTZ concentrations up to 3 μM for 24 h did not result in any significant changes in cell morphology. Cells continued to grow and cultures formed confluent lawns after 72 h. Starting at 6 μM MTZ, however, cells became rounder than usual and were slightly bigger in size. Cells also took longer to attach to surfaces and for cultures to form confluent lawns. Cell viability, however, was verified by cell adhesion and trypan blue exclusion assay. After 29 weeks, cells growing at 12 μM MTZ (MTZR) were obtained. We tried to increase the concentration of MTZ further, but this resulted in further increase in doubling time.

**Figure 1:**
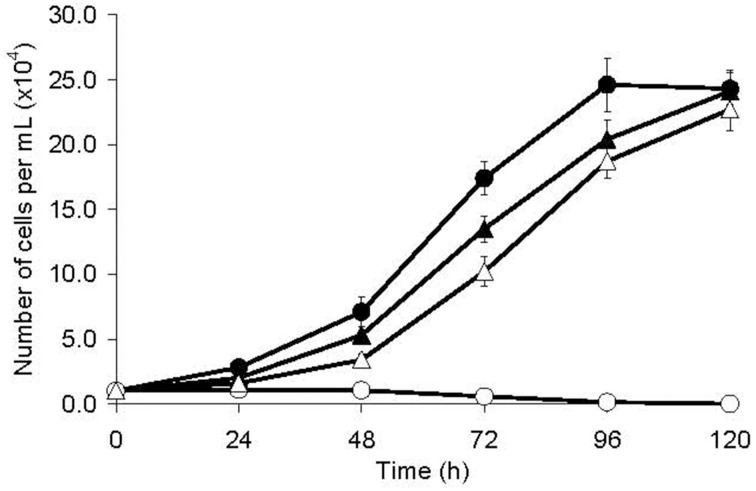
**Growth kinetics of HM-1 (circles) and MTZR (triangles) cultured with (open symbols) or without (filled symbols) 12 μM MTZ**.

The population doubling time of MTZR was significantly longer compared to HM-1 (26.8 ± 0.9 and 18.2 ± 0.5 h, respectively; *P*-value < 0.001, Figure 1). MTZR also appeared rounder, bigger, and more granular than HM-1. In contrast, parental cells exposed to 12 μM MTZ showed signs of morphological changes as early as 2 h, rounding in 5 h, cell shrinkage, and detachment within 24 h. The slight enlargement of MTZR (+) compared to HM-1 was suggested when we measured the total protein content of MTZR and HM-1, 4.81 ± 0.04 and 4.24 ± 0.03 pg/cell, respectively. Flow cytometric analysis also confirmed that MTZR was slightly larger than HM-1 (data not shown).

The IC_50_ of MTZ in MTZR and HM-1 was 12.9 ± 0.3 and 6.5 ± 0.3 μM, respectively, and its resistance index was 2.0 (Table 1). When MTZR was cultured without the drug for 1 week followed by MTZ challenge for 24 h, percent survival was only 28%, indicating that resistance to the drug was reversible (data not shown). To determine if MTZR was cross-resistant with other amebicides, the IC_50_ of the drugs listed in Table 1, against MTZR and HM-1 was determined. Results showed that MTZR had reduced sensitivity to the drugs.

**Figure 2:**
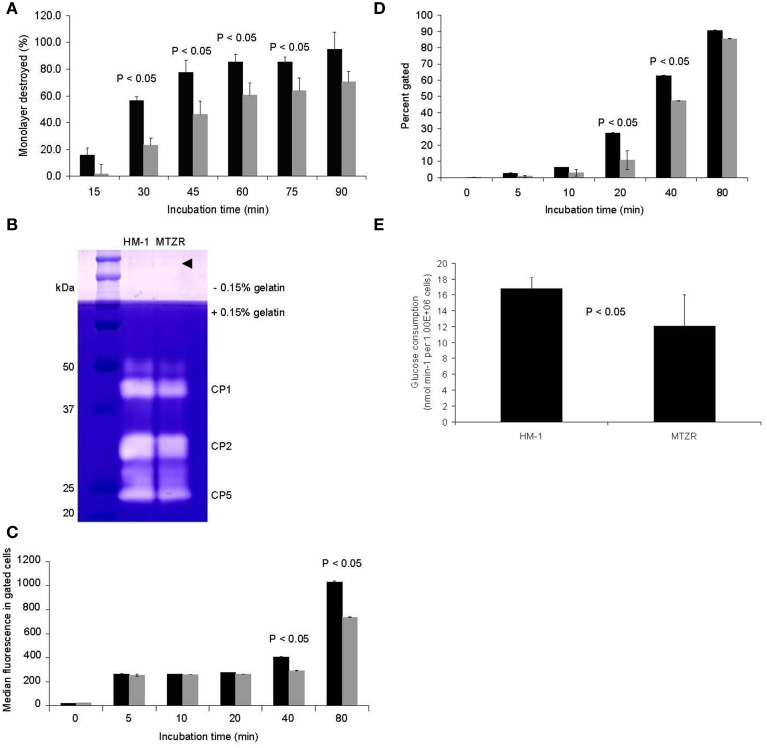
**Phenotypic changes observed in MTZR**. Statistical significance is indicated (*P*-value < 0.05). **(A)** CHO monolayer destruction assay of HM-1 (black bars) and MTZR (gray bars). **(B)** Zymography of whole cell lysates of HM-1 and MTZR. An arrowhead indicates comparable protein loading per lane as estimated by CBB staining of irrelevant proteins. **(C,D)** Phagocytosis assay with carboxylated beads in HM-1 (black bars) and MTZR (gray bars). The number of beads phagocytosed **(C)** and the percentage of cells with engulfed beads **(D)** at indicated time points are compared between HM-1 and MTZR. **(E)** Glucose consumption assay.

MTZR had a slower rate of CHO monolayer destruction, especially at early time points, indicating a slight decrease in virulence (*P*-value < 0.05 for 30, 45, 60, and 75 min; Figure 2A). This was consistent with the observed decrease in intensity of bands corresponding to cysteine protease 1 and 2 (CP1 and CP2, synonymous to EhCP-A1 and EhCP-A2, respectively) in the zymogram (Figure 2B), and by an ImageJ (http://rsbweb.nih.gov/ij/) analysis of the same bands that indicated an approximately 33% decrease in band areas. The band for CP5 (EhCP-A5) and control bands (arrow head) were comparable between HM-1 and MTZR. The decrease in cytopathy in MTZR was also indicated by the lower the number of engulfed beads (Figure 2C) and percentage of cells with engulfed beads in the phagocytosis assay (Figure 2D). MTZR also consumed glucose at a reduced rate compared to HM-1 (*P*-value < 0.05, Figure 2E). In addition, MTZR adhered to plastic surfaces, and to fibronectin- and collagen-coated plates at relatively comparable levels, but not as efficiently as HM-1 (Supplementary Figure S2).

### Transcriptomic analysis of MTZR

**Figure 3:**
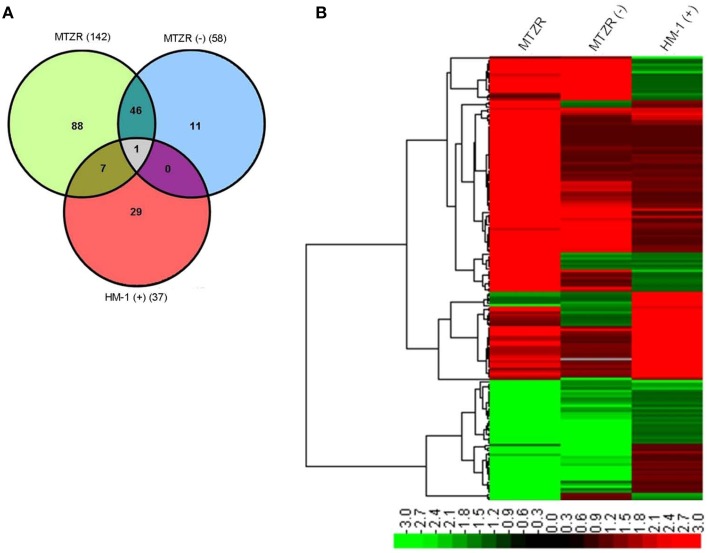
**Venn diagram (A) and heat map (B) of modulated genes in MTZR, MTZR (−), and HM-1 (+)**. Note that most of genes that were transcriptionally regulated in MTZR were specific to the strain when cultured with MTZ.

Our expression data showed variations in the number of differentially expressed genes between MTZR and HM-1 cultured with and without MTZ (GEO ID: GSE35990). To identify genes whose expression was most highly affected, a general filtering of the expression data, as indicated in Materials and Methods, was used. This resulted in the identification of 142 probe sets for MTZR, 58 probe sets for MTZR (−), and 37 probe sets for HM-1 (+) that showed at least 3-fold changes compared to HM-1 (−) (Figure 3A, Table S3). In MTZR, 95 (66.9%) of the genes were upregulated, while 47 (33.1%) genes were downregulated (Tables 2, 3). Only 55 (38.7%) genes were annotated after conducting BLAST search, while 87 (61.3%) genes encoded for proteins of unknown function, i.e., hypothetical proteins (Table S4). The most upregulated gene was a hypothetical protein (HP) (EHI_006850) with some similarity to a zinc finger protein in *E. histolytica* (*E*-value = 1.0e^−46^), designated as HP1 in this study. Its expression in MTZR was increased by 36.9 folds. Genes for DNA polymerase (EHI_164190), iron-sulfur flavoprotein (ISF) (EHI_025710), AIG1 family protein [XP_648192 (522.m00018)], and four other HPs were also upregulated by more than 10 folds. The most downregulated genes were three leucine-rich proteins [EHI_033560, EHI_077280, EHI_124070 (371.m00031)] which had fold changes of more than 160, and two AIG family proteins [XP_648014 (628.m00011), EHI_180390] and two CPs (EHI_160330, EHI_121160) genes that were downregulated by at least 5 folds. The two CPs identified had high similarity to CP7 (synonymous to EhCP-B1) (81%) based on amino acid sequence (Tillack et al., 2007). The differential expression of 16 genes was validated by qRT-PCR, using rpoII as an endogenous control (Table 4). Pearson's correlation coefficient (0.81) between microarray and qRT-PCR results indicated a reasonable degree of concordance between the fold-changes of the genes selected for this comparison.

**Table 2:** **List of annotated genes that were upregulated in MTZR by >3 fold compared to HM-1**.

**Probe Set ID**	**NCBI RefSeq**	**Gene name**	***P*-value**	**Fold-change**
EHI_164190_at	XM_001913665.1	DNA polymerase, putative	2.80E-03	12.9
EHI_025710_at	XM_644279.1	Iron-sulfur flavoprotein, putative	4.50E-05	11.6
522.m00018_at	XM_643100.1	AIG1 family protein	6.50E-04	11.5
EHI_189960_at	XM_647238.1	ADP-ribosylation factor 1, putative	9.20E-04	7.2
EHI_026000_s_at	XM_643099.1	AIG1 family protein, putative	2.60E-03	6.5
EHI_138480_at	XM_650038.1	Iron-sulfur flavoprotein, putative	6.50E-04	6.3
EHI_073980_s_at	XM_648468.1	Serine-rich 25 kDa antigen protein	1.10E-02	6.2
EHI_022270_s_at	XM_644761.1	Iron-sulfur flavoprotein	1.60E-02	5.5
EHI_129890_at	XM_646723.1	Type A flavoprotein, putative	1.00E-02	5.4
EHI_022600_s_at	XM_643169.1	Iron-sulfur flavoprotein	1.40E-02	5.4
EHI_181710_s_at	XM_001914510.1	Iron-sulfur flavoprotein, putative	1.30E-02	5.3
82.m00157_s_at	XM_648374.1	Surface antigen ariel1	6.80E-03	5.3
EHI_096770_at	XM_650580.1	Acetyltransferase, putative	1.30E-02	4.9
EHI_072960_s_at	XM_001914217.1	dUTP nucleotidohydrolase domain protein	3.20E-02	4.9
EHI_067720_s_at	XM_643101.1	Iron-sulfur flavoprotein, putative	5.30E-04	4.8
EHI_072000_s_at	XM_001913846.1	Serine-rich 25 kDa antigen protein, putative	9.40E-03	4.8
EHI_103260_s_at	XM_001913434.1	Iron-sulfur flavoprotein	2.30E-02	4.5
82.m00164_s_at	XM_648353.1	Serine-rich 25 kDa antigen protein	4.10E-02	4.1
EHI_074750_at	XM_644490.1	Ras family GTPase	3.80E-04	3.9
EHI_148550_at	XM_652392.1	Protein tyrosine kinase domain-containing protein	4.10E-02	3.8
432.m00028_at	XM_643464.1	AIG1 family protein	2.70E-04	3.7
EHI_075660_at	XM_643678.1	CAAX prenyl protease, putative	1.70E-04	3.6
EHI_075150_at	XM_643772.2	NAD-specific glutamate dehydrogenase, putative	6.10E-03	3.6
EHI_126550_at	XM_643463.2	AIG1 family protein, putative	2.00E-03	3.5
EHI_082060_at	XM_646822.1	Leucine rich repeat protein, BspA family	1.00E-02	3.4
36.m00218_s_at	XM_649994.1	Truncated ABC transporter, putative	4.80E-02	3.3
EHI_147020_at	XM_644619.1	Ser/thr protein phosphatase family protein	9.30E-04	3.3
EHI_092100_at	XM_649470.1	Chitinase, putative	4.10E-02	3.2
EHI_045450_at	XM_652204.1	Ras family GTPase	8.70E-04	3.1
EHI_029620_s_at	XM_642949.1	Aldose reductase, putative	1.20E-03	3.1
EHI_118410_at	XM_644811.1	Tyrosine kinase, putative	7.20E-03	3.1
EHI_075640_at	XM_001914030.1	Protein phosphatase domain-containing protein	2.10E-02	3.1
EHI_045600_at	XM_648507.1	Ras family protein	2.30E-02	3.1
EHI_126560_at	XM_001914189.1	AIG1 family protein, putative	1.40E-02	3.1
EHI_091450_at	XM_645308.2	Cysteine protease, putative	9.00E-04	3.0

**Table 3:** **List of annotated genes that were downregulated in MTZR by > 3 fold**.

**Probe Set ID**	**NCBI RefSeq**	**Gene name**	***P*-value**	**Fold-change**
EHI_033560_s_at	XM_001913757.1	Leucine rich repeat protein 1	2.20E-04	171.4
EHI_077280_s_at	XM_649853.2	Leucine rich repeat protein, BspA family	1.70E-04	163.1
371.m00031_s_at	XM_643815.1	BspA-like leucine rich repeat protein, putative	3.50E-04	163
628.m00011_at	XM_642922.1	AIG1 family protein	4.10E-04	8.4
EHI_160330_s_at	XM_001914054.1	Cysteine protease, putative	3.90E-03	7.3
EHI_121160_s_at	XM_001914417.1	Cysteine protease, putative	2.20E-03	7.3
EHI_054690_at	XM_646973.2	Metal dependent hydrolase, putative	4.90E-04	5.8
EHI_180390_at	XM_648725.1	AIG1 family protein, putative	2.40E-03	5.0
EHI_174230_s_at	XM_647412.2	S-adenosylmethionine synthetase	5.40E-03	4.1
EHI_176700_at	XM_001914268.1	AIG1 family protein, putative	1.00E-02	4.1
EHI_179060_at	XM_651187.2	Glycosyltransferase	9.10E-03	3.6
EHI_176580_at	XM_643164.1	AIG1 family protein, putative	5.90E-04	3.5
EHI_090260_at	XM_645039.1	Competence/damage-inducible protein, putative	6.80E-04	3.4
EHI_020250_at	XM_643256.1	Lecithin:cholesterol acyltransferase protein	7.30E-03	3.3
2.m00624_s_at	XM_652243.1	Protein kinase, putative	3.20E-02	3.3
EHI_006140_at	XM_648345.1	Rho guanine nucleotide exchange factor, putative	3.00E-02	3.2
EHI_026360_s_at	XM_650291.1	Phosphoserine aminotransferase, putative	6.30E-03	3.2
554.m00020_s_at	XM_643035.1	AIG1 family protein, putative	3.00E-03	3.0
EHI_067220_at	XM_647568.1	Rho family GTPase	1.90E-03	3.0
EHI_061760_at	XM_643432.1	Protein folding regulator	2.00E-02	3.0

**Table 4:** **qRT-PCR validation of selected genes from our microarray data**.

**Probe set ID**	**Gene name**	**Fold change by Microarray**	**Fold change by qRT-PCR**	**Regulation**
EHI_096770_at	Acetyltransferase, putative	4.9	4.6	Upregulated
EHI_176700_at	AIG1 family protein, putative	4.1	5.1	Downregulated
EHI_126550_at	AIG1 family protein, putative	3.5	5.1	Upregulated
EHI_164190_at	DNA polymerase, putative	12.9	2.5	Upregulated
EHI_006850_at	Hypothetical protein 1	36.9	57.8	Upregulated
EHI_165190_at	Hypothetical protein 2	27.1	16.3	Upregulated
EHI_127670_at	Hypothetical protein 3	17	25.2	Downregulated
EHI_087210_at	Hypothetical protein 4	8.9	22.5	Upregulated
EHI_054690_at	Metal dependent hydrolase, putative	5.8	15.4	Downregulated
EHI_138480_at	Iron-sulfur flavoprotein, putative (ISF1)	6.3	5.8	Upregulated
EHI_025710_at	Iron-sulfur flavoprotein, putative (ISF2)	11.6	15.8	Upregulated
EHI_129890_at	Type A flavoprotein, putative	5.4	3.8	Upregulated
EHI_022600_s_at	Iron-sulfur flavoprotein (ISF4)	5.4	8.1	Upregulated
EHI_075150_at	NAD-specific glutamate dehydrogenase, putative	3.6	16.8	Upregulated
EHI_147020_at	Ser/thr protein phosphatase family protein	3.3	1.9	Upregulated
EHI_118410_at	Tyrosine kinase, putative	3.1	2.3	Upregulated

To determine whether the genes we identified were important in MTZ resistance or resulted from a general stress response, a 3-way comparison of differentially expressed genes in MTZR, MTZR (−) and HM-1 (+) was performed. Figures 3A,B, and Table S3 show that out of 142 genes modulated in MTZR, 48 genes (33.8%) were also modulated in MTZR (−), while only 8 genes (5.6%) were differentially transcribed in HM-1 (+). Among the genes specifically modulated in MTZR were DNA polymerase (EHI_164190), 4 AIG1 family proteins [EHI_026000, EHI_126550, EHI_126560, XP_648127 (554.m00020)], four iron-sulfur flavoproteins (EHI_022270, EHI_022600, EHI_181710, EHI_067720, EHI_103260), metal-dependent hydrolase (EHI_054690), dUTP nucleotidohydrolase domain-containing protein (EHI_072960), three serine-rich 25 kDa antigen protein [EHI_073980, EHI_072000, EHI_072000 (82.m00164)], two Ras family protein (EHI_074750, EHI_045600), Rho guanine nucleotide exchange factor (EHI_006140), and a competence/damage-inducible protein (EHI_090260). In MTZR (−), AP-2 complex protein (EHI_014390), serine acetyltransferase (EHI_021570), zinc finger protein (EHI_091050), cysteine protease (EHI_117650), and a cell division control protein (EHI_154270) were specifically modulated. In HM-1 (+), on the other hand, genes for heat shock proteins [EHI_005657 (181.m00064), EHI_156560 (482.m00014), EHI_034710)] were specifically upregulated together with genes putatively involved in drug resistance like P-glycoprotein 5 (EHI_075410, EHI_125030) and ATP-binding cassette protein (EHI_134470, EHI_178580). Two iron-sulfur flavoproteins (EHI_025710, EHI_138480) and an ABC transporter [EHI_084730 (36.m00218)] were upregulated in both MTZR and HM-1 (+), although their expression in HM1 (+) was 2 to 3 folds higher. It is also interesting to note that in all three strains, induction of gene transcription clearly prevailed over inhibition. This was particularly true for HM-1 (+) where almost 95% of the modulated genes were upregulated.

### Go classification, features, and clustering on the genome

To have a general view of the cellular functions regulated as a result of MTZ resistance, we performed a GO enrichment analysis. A total of 132 GO terms were mined from Amoeba DB representing 63 unique GO IDs (Table S5). However, based on Fisher's exact test, only 24 terms were significantly enriched in MTZR (Table 5). Genes involved in GTP binding and proteolysis were the most highly enriched, followed by genes associated with cysteine-type peptidase activity, small GTPase-mediated signal transduction, oxidoreductase activity, protein kinase activity, and electron carrier activity. Roughly, the enriched terms could be classified into four functional categories, namely (1) nucleotide binding, (2) metabolism, (3) oxidative stress response, and (4) signal transduction.

**Table 5:** **List of GO terms that were significantly enriched in MTZR**.

**GO ID**	**GO Name**	**Aspect**	**Count**	***p*-value**
GO:0005525	GTP binding	Function	14	3.95E-06
GO:0005622	Intracellular	Component	11	8.89E-03
GO:0006508	Proteolysis	Process	10	2.78E-08
GO:0007264	Small GTPase mediated signal transduction	Process	8	4.58E-05
GO:0008234	Cysteine-type peptidase activity	Function	8	5.09E-09
GO:0016491	Oxidoreductase activity	Function	7	2.06E-05
GO:0008152	Metabolic process	Process	6	5.52E-03
GO:0004713	Protein tyrosine kinase activity	Function	5	5.20E-10
GO:0006508	Proteolysis	Process	4	1.34E-02
GO:0009055	Electron carrier activity	Function	4	9.26E-05
GO:0015031	Protein transport	Process	3	3.99E-03
GO:0003887	DNA-directed DNA polymerase activity	Function	2	2.06E-02
GO:0004222	Metalloendopeptidase activity	Function	2	1.54E-03
GO:0005089	Rho guanyl-nucleotide exchange factor activity	Function	2	4.95E-02
GO:0005886	Plasma membrane	Component	2	5.95E-04
GO:0006260	DNA replication	Process	2	2.66E-02
GO:0006520	Cellular amino acid metabolic process	Process	2	1.84E-03
GO:0008408	3′-5′ exonuclease activity	Function	2	7.91E-04
GO:0010181	FMN binding	Function	2	3.29E-03
GO:0035023	Regulation of Rho protein signal transduction	Process	2	4.95E-02
GO:0004648	O-Phospho-L-serine:2-oxoglutarate aminotransferase activity	Function	1	5.39E-03
GO:0006564	L-Serine biosynthetic process	Process	1	5.39E-03
GO:0006913	Nucleocytoplasmic transport	Process	1	5.39E-03
GO:0008483	Transaminase activity	Function	1	3.71E-02
GO:0050662	Coenzyme binding	Function	1	4.23E-02

Only a fraction of the differentially expressed genes in MTZR (about 5%) encoded for membrane proteins, based on positive results for transmembrane and signal peptide predictions (Table S6). Only 21 genes (14.8%) contained at least one transmembrane domain. Eleven of these were upregulated, while 10 were downregulated. Proteins encoded by 25 genes (17.6%) also had predicted signal peptides. Only seven genes have both transmembrane domains and signal peptides. Only one, however, was annotated as tyrosine kinase (EHI_118410), while the rest were all genes for HPs.

Most of the modulated genes were encoded on different scaffolds. Thirty-four genes (30.6% of EHI-probe sets), however, formed 15 sets of dyads or triads that occupied the same contig (Table S7). Included were genes for four AIG1 family proteins (EHI_126550, EHI_126560, EHI_176700, EHI_176580) and two Ras family proteins (EHI_045450, EHI_045600). In four sets of genes, the members were found to be located on opposite strands, but the regulation of their expression was the same and their fold-changes were comparable. For some genes found on the same strand, the distance between the genes was less than 1 kb. It was therefore possible for these genes to be co-regulated by the same transcription factor.

### Functional analysis

**Figure 4:**
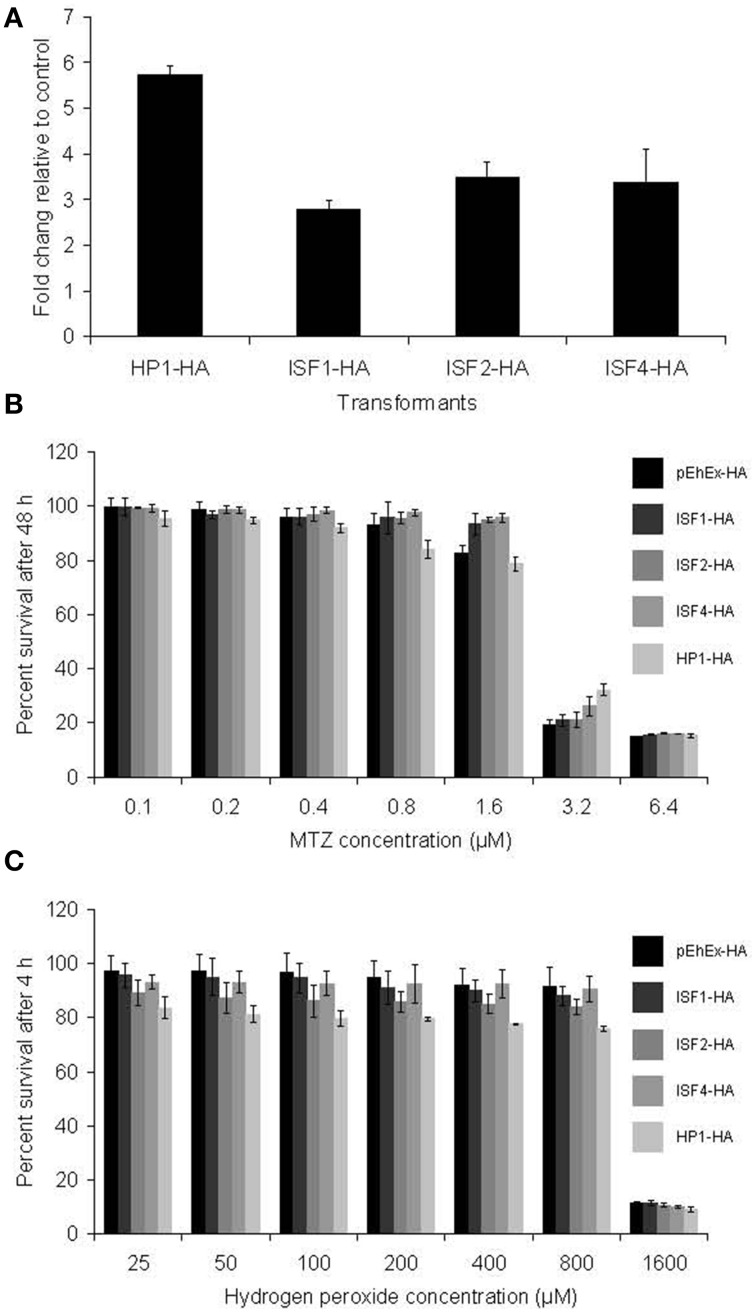
**(A)** qRT-PCR measurement of transfected genes in HP1-HA, ISF1-HA, ISF2-HA, and ISF4-HA showed overexpression of the genes. Fold changes relative to pEhEx-HA are shown. **(B)** MTZ challenge of the transformants. Transformants cultured with 10 μM G418 were challenged with MTZ for 48 h. **(C)** Hydrogen peroxide challenge of the transformants. Indicated transformants were cultured with 25–1600 μM hydrogen peroxide for 4 h.

To study further the relationship between the expression of some of the modulated genes in MTZR with drug resistance, generation of stable transformants overexpressing HA-tagged HP1, ISF1, ISF2, and ISF4 was performed (Figure 4A). The mRNA level of these proteins was estimated by qRT-PCR. The levels of mRNA of HP1 and ISFs were about 5.5 or 2.5 to 3 fold, respectively, higher in MTZR compared to their expression in HM-1 transfected with mock vector (pEhEx-HA). Transformants cultured with 10 μM G418 were challenged with MTZ for 48 h (Figure 4B). The highest concentration shown was based on the IC_50_ of MTZ against HM-1, performed under the same conditions (6.5 μM). No significant difference in percent survival was observed at all concentrations tested between the overexpressors and control (Figure 4B). Neither was significant difference in survival against hydrogen peroxide, observed, among the transformants (Figure 4C).

## Discussion

### Differences in MTZR generation in this study and previous works

Currently, only 4 studies have been published regarding *E. histolytica* and its response to or resistance to MTZ (Samarawickrema et al., 1997; Wassmann et al., 1999; Leitsch et al., 2007; Tazreiter et al., 2008). These studies either exposed wild-type cells to serum level concentrations of the drug (70–100 μM) or generated drug resistant trophozoites, similar to what we did in this study. However, the MTZ concentrations used in these studies and ours differ, which might explain some of the differences observed. The concentration used here is slightly higher compared to those used by Samarawickrema et al. 10 μM (Samarawickrema et al., 1997), but significantly lower than those used by Wassmann et al. 40 μM (Wassmann et al., 1999), Tazreiter et al. 50 μM (Tazreiter et al., 2008), and Leitsch et al. 50 μM (Leitsch et al., 2007). Our strain adapted more quickly to 1 μM MTZ compared to the strain used by Samarawickrema (7 vs. 38 days) but overall the length of time used to generate MTZR was comparable.

For reasons we do not understand, our attempts to further increase the level of resistance failed, and no resistance above the concentration used here could be established. We could not culture MTZR consistently at drug concentrations higher than 12 μM. In contrast, strains of *Trichomonas* and *Giardia* can be readily adapted to grow at 584 μM and 115 μM MTZ, respectively (Kulda et al., 1984; Townson et al., 1992). Our results are therefore consistent with previous reports indicating that MTZ resistant strains are more difficult to generate in *E. histolytica* (Samarawickrema et al., 1997). While the concentration we used is several times lower than serum levels after MTZ treatment (Van Oosten et al., 1986), the changes we observed may still provide insights as to what occurs in the parasite *in vivo*. It was previously reported that in an abscess, parasites encounter significantly lower levels of drug than those found in the serum as drug penetration is limited by poor perfusion and mechanical barriers such as fibrin clots and the abscess wall (Sirinek, 2000).

### Decrease in growth and reversibility of resistance in MTZR

Formation of drug resistance is often accompanied by fitness costs (Andersson and Hughes, 2010). One particular cost, reduction in growth rate, is the main parameter that determines the rate by which resistance develops and level of resistance reached. Similar to resistant strains previously generated (Samarawickrema et al., 1997; Wassmann et al., 1999), the doubling time of MTZR was longer compared to HM-1. Fitness cost in growth associated with drug resistance are well documented in mammalian cells, e.g., lymphoma and cancer cells (Lee, 1993; Bishop et al., 2001). It should be noted, however, that the growth kinetics of MTZR in the absence of drug pressure improved over time. This indicates that some of the drug-selection-induced changes developed by MTZR were temporal and reversible. This also indicates that cells in the culture may have varying degrees of resistance. In addition, MTZR (−) that was re-exposed to MTZ showed significantly lower growth compared to MTZR, but slightly higher compared to HM-1, which may indicate that some drug-selection-induced changes in MTZR were still operative even after the removal of drug pressure.

### Cross-resistance in MTZR

The resistance level displayed by MTZR is significantly lower compared to previous reports: the IC_50_ value of 12 μM in this study compared to 40 μM in the previous study (Samuelson, 1999; Wassmann et al., 1999). However, its survival in otherwise lethal concentration of MTZ for long periods of incubation, does indicate reduced sensitivity and resistance. The cross-resistance of MTZR to ornidazole and tinidazole is not surprising, because both are 5-nitroimidazoles like MTZ. It is therefore likely that their mode of action and mechanism of resistance were similar (Pasupuleti et al., 2014). *T. vaginalis* isolates that were characterized as having very high resistance to MTZ were similarly insensitive to tinidazole (Narcisi and Secor, 1996). In the case of paromomycin, emetine, chloroquine, and hydrogen peroxide, it is possible that the reduced sensitivity was nonspecific and could be due to an increased capacity of MTZR to deal with stress. Studies have shown that cell lines resistant to one drug are often cross-resistant to closely related drugs or to unrelated compounds when their mode of action is the same or when they carry membrane alterations (Upcroft and Upcroft, 1993).

### Decreased adhesion, phagocytosis, cytolysis, and virulence in MTZR

In *E. histolytica*, establishment and outcome of infection depend heavily on adhesive and invasive capacity (Flores-Romo et al., 1997). Recently, drug resistance has been shown to modulate these factors and vice versa (Giha et al., 2006). The decreased rate of adhesion observed in MTZR indicated that MTZ selection negatively affected the synthesis or expression of surface adhesion molecules. While other studies have shown that cell adhesion is a key determinant in drug resistance (Shain and Dalton, 2001), this apparently was not the case in MTZ resistance. Inhibition of adherence in this parasite has previously been shown to decrease host cell cytotoxicity (Ravdin et al., 1985) and experimental evidence strongly suggests the involvement of surface adhesins in phagocytosis (Heron et al., 2011). As shown in the results, MTZR also had decreased cytopathy and phagocytosis (Figures 2A,C). The decreased band intensity for CP1 and CP2 in the zymogram may partially explain this phenotype. CP1 has been shown to be upregulated in a mouse model of amebic colitis following invasion (Gilchrist et al., 2006), while CP2 has been reported to contribute to intestinal damage and liver abscess formation (Hellberg et al., 2001). Our microarray data, however, did not show any significant repression of these genes, except for two uncharacterized CPs that were repressed by more than 7 folds. These CPs had high similarity (81%) to CP7 (also called EhCP-B1) based on amino acid sequence (Tillack et al., 2007). No significant change was observed in the expression of the genes known to be involved in the inactivation and intracellular trafficking of CPs such as intrinsic inhibitors of CPs (Sato et al., 2006) and cysteine protease binding protein family 1 (Nakada-Tsukui et al., 2012) and other lysosomal hydrolase carriers (Furukawa et al., 2012, 2013; Marumo et al., 2014). It is also possible, however, that resistance to MTZ may not necessarily result to decreased virulence *in vivo*.

### Implications from transcriptomic profiling of MTZR

Our transcriptomic profiling suggests that the transcriptomic changes we observed in MTZR strain cultured with MTZ were a combination of the changes responsible for MTZ resistance and those associated with the adaptation to stresses caused by the exposure to MTZ. However, we believe that among the observed changes, the changes that were specific to MTZR strain with MTZ and absent in HM-1 with MTZ were responsible, at least in part, for resistance, rather than adaptation. This is based on the following observations: the transcriptomic profile of *E. histolytica* HM-1 cultured with MTZ was remarkably different from that of MTZR strain cultured with MTZ, while MTZR strain cultured with or without MTZ showed similar transcriptomic profiles. In other words, a larger number of common changes in gene expression were identified between MTZR strain cultured with MTZ and those without MTZ when compared between MTZR strain cultured with MTZ and HM-1 cultured with MTZ.

GO enrichment of MTZR-associated transcriptomic changes revealed regulation of genes encoding for proteins potentially involved in nucleotide binding, metabolism, oxidative stress response, and signal transduction. These are apparently the processes most influenced by MTZ resistance. However, since most of the genes are without GO annotations, it is likely that additional processes are involved. When we examined the genomic locations of these genes, we found that 30% of them formed dyads or triads that occupied the same contig. While some genes forming dyads and triads are apart by more than 10 kb, the distance between some of the adjacent genes in dyads and triads was less than 1 kb. Thus, co-expression of some genes in dyads and triads in MTZR could be attributed to a shared regulatory system.

Consistent with previous findings, no significant difference in PFOR mRNA levels was observed between MTZR and HM-1 (Samarawickrema et al., 1997; Wassmann et al., 1999; Tazreiter et al., 2008). This observation could mean 3 things. First, it's likely that the level of resistance achieved in this study was not sufficient to downregulate PFOR, although a similar finding was reported in strains resistant to 40 μM MTZ (Wassmann et al., 1999). Second, since *E. histolytica* lacks pyruvate dehydrogenase (Clark et al., 2007; Tazreiter et al., 2008) or pyruvate decarboxylase (Lo and Reeves, 1978), the significance of PFOR cannot be overstated. The lack of an alternative enzyme to compensate for its downregulation makes this enzyme difficult to repress without severe consequences to the ameba. Third, in contrast to *Giardia* and *Trichomonas*, lack of PFOR downregulation may indicate the existence of an alternative mechanism for MTZ resistance in this parasite. It should be noted, however, that clinically resistant isolates of *Trichomonas* have been reported with no decrease in PFOR activity (Müller and Gorrell, 1983).

Two previous studies have reported the upregulation of Fe-SOD and downregulation of Fdx in the MTZ resistant ameba (Samarawickrema et al., 1997; Wassmann et al., 1999). In the previous work, fold changes were determined based on enzyme activity assay or northern blot (Samarawickrema et al., 1997; Wassmann et al., 1999). Disparity, however, between changes in mRNA abundance and enzyme activity have been reported, and in some cases, increases in enzyme activity can exceed those of mRNA abundance (Glanemann et al., 2003). Interestingly, the lack of Fe-SOD modulation in cells exposed to MTZ was also reported by Tazreiter et al. (2008). On the contrary, in this work, neither gene was modulated significantly. It is possible that similar to PFOR, differences in resistance levels could explain the lack of modulation in the levels of Fe-SOD and Fdx. Other studies also indicated that Fe-SOD and Fdx are not tightly associated with MTZ resistance. It was shown that parasites in which Fdx was repressed did not display full resistance to MTZ (Rasoloson et al., 2002) and in bacteria, increased SOD activity was not always associated with MTZ resistance (Smith and Edwards, 1995).

In this study, genes involved in nucleic acid synthesis and binding, stress response, metabolism, and vesicular trafficking were mostly affected in MTZR. The significance of these genes relative to phenotypic changes and drug resistance will be discussed here through their known roles in *E. histolytica* or in other organisms, in regulating key cell functions. One should also note that since only a single MTZ resistant line was analyzed in this study, it is possible that MTZ resistance can be also conferred by the mechanisms that are independent of those described in this study.

#### Nucleic acid synthesis and binding

In prokaryotes, upregulation of DNA polymerases imparts plasticity to their genome, allowing them to adapt in unfavorable environmental conditions (Joseph et al., 2006). It has been suggested that pathogenic bacteria utilize this adaptation to develop resistance against therapeutic agents (Karpinets et al., 2006). Since it has been shown that primary effect of MTZ is rapid inhibition of DNA replication (Ludlum et al., 1988; Sisson et al., 2000; Leitsch et al., 2007), the upregulation of a DNA polymerase (EHI_164190) in this study may indicate that this mode of action is operative in MTZ resistance. One hypothetical protein identified in this study (HP1, EHI_006850) has some similarity to zinc finger proteins, which are known to be involved in DNA recognition, RNA packaging, and transcriptional activation (Laity et al., 2001). It has an NAD+ binding pocket and a tetrachlorodibenzo-p-dioxin (TCDD)-inducible poly(ADP-ribose) polymerase (PARP)-like domain that is involved in the attachment of ADP-ribose units to DNA-binding proteins (Ma et al., 2001). It's also known to be a regulatory component induced by DNA damage and an enzyme involved in DNA repair (Nguewa et al., 2006; Marchler-Bauer et al., 2011). The dUTP nucleotidohydrolase (EHI_072960) upregulated in MTZR may be involved in the removal of dUTP from the dNTP pool, *de novo* biosynthesis of dTTP, and DNA replication as reported (Richards et al., 1984; Gadsden et al., 1993). In cancer cells, high level of dUTPase is implicated as a mechanism of resistance to chemotherapy (Ladner et al., 2000).

#### Stress response

ISFs that were modulated in MTZR are part of family of redox-active proteins mainly found in anaerobic prokaryotes (Andrade et al., 2005). In NCBI, 18 *ISF* genes are annotated in the *E. histolytica* genome, although 50% of these are identical proteins (http://www.ncbi.nlm.nih.gov/). In this study 7 ISF probe sets were upregulated by at least 4 folds in MTZR. The overexpression of ISF1 (EHI_138480) was also observed in HM-1 (+), while ISF2 was upregulated in both MTZR (−) and HM-1 (+). These 2 ISFs were upregulated in HM-1 (+) by least 20 folds, compared to 6 to 11 folds in MTZR, hinting that these genes are involved in stress response. These two genes were also induced in *E. histolytica* cultured under L-cysteine deprived and oxidative stress conditions (Vicente et al., 2009; Husain et al., 2011), but were downregulated in a mouse model of intestinal amebiasis (Gilchrist et al., 2006). Our findings together with these reports suggest that induction of these ISFs, particularly ISF1, is an adaptation that may enhance survival when cells experience environmental stress.

#### Metabolism

Several metabolic genes were upregulated in MTZR. NAD-specific glutamate dehydrogenase (EHI_075150) catalyzes the oxidative deamination of glutamate to α-ketoglutarate using NAD as cofactor (Plaitakis and Zaganas, 2001; Girinathan et al., 2014). Most likely, its upregulation is a part of stress response. In humans, its activity is regulated through ADP-ribosylation, and interestingly, Arf1 (EHI_189960) was also upregulated in this study (Herrero-Yraola et al., 2001). Chitinase (EHI_092100) (Escueta- De Cadiz et al., 2013) was also upregulated in MTZR and MTZR (−) by at least 3 folds, although its precise role in drug resistance is not known. In plants, its upregulation plays a defensive role against toxins and antibiotics (Gooday, 1999). Aldose reductase (EHI_029620), on the other hand, can reduce a broad spectrum of substrates including cytotoxic aldehydes (Ramana, 2011). Its upregulation in human liver cancer cells was associated with resistance to daunorubicin and was confirmed by sensitizing cells to the drug by the addition of aldose reductase inhibitors (Lee et al., 2001).

The decrease in cell growth observed in MTZR may be partially explained by the repression of phosphoserine aminotransferase (EHI_026360) (Ali and Nozaki, 2006; Mishra et al., 2010), which catalyzes the conversion of 3-phosphohydroxypyruvate to L-phosphoserine, the second step of phosphorylated serine biosynthetic pathway (Ali and Nozaki, 2006). L-serine has a well-recognized role in cell proliferation, providing precursors for amino acids, protein synthesis, and nucleotide synthesis (Nozaki et al., 2005; Tabatabaie et al., 2010). The downregulation of this enzyme, therefore, may contribute to the decreased growth rate. The repression of lecithin: cholesterol acyltransferase (EHI_020250) may also be associated with decreased growth rate, because the enzyme is crucial in the maturation, remodeling, and metabolism of high density lipoprotein, HDL (Calabresi et al., 2011).

#### BspA1 gene family

Several genes encoding for leucine-rich repeat (LRR) proteins of the BspA family were also modulated in MTZR, and were the most downregulated genes with fold changes of more than 150. LRR is known to provide a structural framework for the formation of protein-protein interactions and serve as recognition motifs for surface proteins (Kobe and Kajava, 2001). BspA family protein was originally identified in *Bacteroides forsythus* and the LRR motifs of the BspA protein have been linked to cell binding to components of the extracellular matrix (Sharma et al., 1998). It is therefore possible that their downregulation may partially explain the reduced adhesion seen in MTZR. It was previously shown that an LRR BspA family protein in *E. histolytica* was located primarily on the plasma membrane, although it did not show whether the protein was involved in surface interactions (Davis et al., 2006).

#### AIG1 gene family

The *E. histolytica* AIG1 gene family consists of 47 members (Biller et al., 2010), and in this study 10 genes were found to be differentially expressed by 3 to 11 folds. Members of this protein family contain a domain found in *Arabidopsis* protein AIG1, which is likely involved in plant recognition and resistance to bacterial pathogens (Reuber and Ausubel, 1996). In *E. histolytica*, however, some AIG proteins appear to be linked with virulence. Comparative genomic analysis of Japanese clinical isolates indicated that an AIG family protein (EHI_176590) and the region around it were uniquely present in KU50, a strain isolated from a diarrheic patient, but absent in a non-pathogenic isolate called KU27 (Nakada-Tsukui et al., unpublished data). Interestingly, the same gene was downregulated by 2.7 folds in MTZR, and two adjacent genes encoding for AIG family proteins (EHI_176580 and EHI_176700) were also repressed by 3 to 4 folds. A previous study also reported the upregulation of an AIG family protein (EHI_180390) in a pathogenic strain of *E. histolytica* (Biller et al., 2010), although here the same gene was downregulated by 5 folds. It is therefore possible that the downregulation of these AIG family proteins might be related to the decrease in MTZR's cytopathogenicity.

Overall, we believe that these transcriptional changes participate in a global regulatory response resulting to the fitness costs and adaptive responses we observed in MTZR. The fact that more genes were upregulated than downregulated also suggests that increasing the amount of cellular components that play roles in stress response, DNA repair and others, is more important in developing low levels of resistance to MTZ than repression of proteins involved in activating the drug. Indeed, the most profound difference we observed between MTZR and HM-1 was the upregulation of genes for several ISF proteins. It should be noted, however, that more than half of the genes reported in this study are hypothetical. It is also likely that the effects of the repressed genes, although fewer in number, are more important in explaining the growth defect.

### Functional analysis of potential resistance genes

In this study, we examined possible role of genes encoding HP1 and several ISF proteins in MTZ resistance. However, their episomal transfection and overexpression did not confer MTZ resistance to the extent observed in MTZR. Thus, at present, direct causal connection between HP1 and ISF overexpression and MTZ resistance has not been demonstrated. It is likely that in *E. histolytica*, resistance to the drug is multi-factorial in nature and requires the global regulation of genes involved in several key cell processes and not just DNA repair and stress response.

In conclusion, here we described the biological state of *E. histolytica* that was selected for MTZ resistance *in vitro*. Phenotypic and transcriptional profiling revealed fitness costs and adaptive responses that we could associate with drug resistance. Some of the consequences of resistance included decreased cell growth and virulence, which have significant implications on host-pathogen interaction and infection outcome. Comparative transcriptional analysis, on the other hand, revealed genes not previously associated with MTZ resistance in this parasite. The list of differentially transcribed genes also did not indicate in MTZR a reduced capacity to activate MTZ, but hinted on adaptations to tolerate the lethal effects of MTZ. Identifying the signaling mediators for the proteins that these genes encode, however, requires further investigation. Finally, while our data showed that high levels of MTZ resistance cannot be readily induced *in vitro*, the data presented here may still help researchers in better understanding the mechanisms involved should clinical cases of high levels of resistance are reported in the future.

### Conflict of interest statement

The authors declare that the research was conducted in the absence of any commercial or financial relationships that could be construed as a potential conflict of interest.

## References

[B1] Agresti A. (1992). A survey of exact inference for contingency tables. Stat. Sci. 7, 131–153 10.1214/ss/1177011454

[B2] Ali V. Nozaki T. (2006). Biochemical and functional characterization of phosphoserine aminotransferase from *Entameba histolytica*, which possesses both phosphorylated and non-phosphorylated serine metabolic pathways. Mol. Biochem. Parasitol. 145, 71–83. 10.1016/j.molbiopara.2005.09.008 16289358

[B3] Andersson D. I. Hughes D. (2010). Antibiotic resistance and its cost: is it possible to reverse resistance? Nat. Rev. Microbiol. 8, 260–271. 10.1038/nrmicro2319 20208551

[B4] Andrade S. L. Cruz F. Drennan C. L. Ramakrishnan V. Rees D. C. Ferry J. G.. (2005). Structures of the iron-sulfur flavoproteins from Methanosarcina thermophila and *Archaeoglobus fulgidus*. J. Bacteriol. 187, 3848–3854. 10.1128/JB.187.11.3848-3854.2005 15901710 PMC1112032

[B5] Benjamini Y. Hochberg Y. (1995). Controlling the false discovery rate: a practical and powerful approach to multiple testing. J. R. Stat. Soc. Ser. B Stat. Methodol. 57, 289–300.

[B6] Biller L. Davis P. H. Tillack M. Matthiesen J. Lotter H. Stanley S. L. Jr.. (2010). Differences in the transcriptome signatures of two genetically related *Entamoeba histlytica* cell lines derived from the same isolate with different pathogenic properties. BMC Genomics 11:63. 10.1186/1471-2164-11-63 20102605 PMC2823695

[B7] Bishop A. J. Kosaras B. Carls N. Sidman R. L. Schiestl R. H. (2001). Susceptibility of proliferating cells to benzo[a]pyrene-induced homologous recombination in mice. Carcinogenesis 22, 641–649. 10.1093/carcin/22.4.641 11285201

[B8] Calabresi L. Baldassarre D. Simonelli S. Gomaraschi M. Amato M. Castelnuovo S.. (2011). Plasma lecithin:cholesterol acyltransferase and carotid intima-media thickness in European individuals at high cardiovascular risk. J. Lipid Res. 52, 1569–1574. 10.1194/jlr.P014977 21596929 PMC3137023

[B9] Clark C. G. Cecilia U. Alsmark M. Hofer M. Saito-Nakano Y. Ali V.. (2007). Structure and content of the *Entamoeba histlytica* genome. Adv. Parasitol. 65, 51–190. 10.1016/S0065-308X(07)65002-7 18063096

[B10] Davis P. H. Zhang Z. Chen M. Zhang X. Chakraborty S. Stanley S. L.. (2006). Identification of a family of BspA like surface proteins of *Entamoeba histlytica* with novel leucine-rich repeats. Mol. Biochem. Parasitol. 145, 111–116. 10.1016/j.molbiopara.2005.08.017 16199101 PMC1382194

[B11] Diamond L. S. Harlow D. R. Cunnick C. C. (1978). A new medium for the axenic cultivation of *Entamoeba histolytica* and other *Entamoeba*. Trans. R. Soc. Trop. Med. Hyg. 72, 431–432. 10.1016/0035-9203(78)90144-X 212851

[B12] Diamond L. S. Mattern C. F. Bartgis I. L. (1972). Viruses of *Entamoeba histolytica*. I. Identification of transmissible virus-like agents. J. Virol. 9, 326–341. 4335522 10.1128/jvi.9.2.326-341.1972 PMC356300

[B13] Dooley C. P. O'Morain C. A. (1988). Recurrence of hepatic amebiasis after successful treatment with metronidazole. J. Clin. Gastroenterol. 10, 339–342. 10.1097/00004836-198806000-00022 2980773

[B14] Durel P. Couture J. Collart P. Girot C. (1960). Flagyl (metronidazole). Br. J. Vener. Dis. 36, 154–162. 10.1136/sti.36.3.154 13725181 PMC1047348

[B15] Escueta- De Cadiz A. Jeelani G. Nakada-Tsukui K. Caler E. Nozaki T. (2013). Transcriptome analysis of encystation in *Entamoeba invadens*. PLoS ONE 8:e74840. 10.1371/journal.pone.0074840 24040350 PMC3770568

[B16] Flores-Romo L. Estrada-García T. Shibayama-Salas M. Campos-Rodríguez R. Bacon K. Martínez-Palomo A.. (1997). *In vitro Entamoeba histlytica* adhesion to human endothelium: a comparison using two strains of different virulence. Parasitol. Res. 83, 397–400. 10.1007/s004360050271 9134567

[B17] Furukawa A. Nakada-Tsukui K. Nozaki T. (2012). Novel transmembrane receptor involved in phagosome transport of lysozymes and β-hexosaminidase in the enteric protozoan *Entamoeba histlytica*. PLoS Pathog. 8:e1002539. 10.1371/journal.ppat.1002539 22383874 PMC3285589

[B18] Furukawa A. Nakada-Tsukui K. Nozaki T. (2013). Cysteine protease-binding protein family 6 mediates the trafficking of amylases to phagosomes in the enteric protozoan *Entamoeba histlytica*. Inf. Immun. 81, 1820–1829. 10.1128/IAI.00915-12 23509141 PMC3648001

[B19] Gadsden M. H. McIntosh E. M. Game J. C. Wilson P. J. Haynes R. H. (1993). dUTP pyrophosphatase is an essential enzyme in *Saccharomyces cerevisiae*. EMBO J. 12, 4425–4431. 8223452 10.1002/j.1460-2075.1993.tb06127.x PMC413740

[B20] Giha H. A. Elbashir M. I. A-Elbasit I. E. A-Elgadir T. M. ElGhazali G. E. Mackinnon M. J.. (2006). Drug resistance-virulence relationship in *Plasmodium falciparum* causing severe malaria in an area of seasonal and unstable transmission. Acta Trop. 97, 181–187. 10.1016/j.actatropica.2005.10.004 16336938

[B21] Gilchrist C. A. Houpt E. Trapaidze N. Fei Z. Crasta O. Asgharpour A.. (2006). Impact of intestinal colonization and invasion on the *Entamoeba histlytica* transcriptome. Mol. Biochem. Parasitol. 147, 163–176. 10.1016/j.molbiopara.2006.02.007 16569449

[B22] Girinathan B. P. Braun S. E. Govind R. (2014). *Clostridium difficile* glutamate dehydrogenase is a secreted enzyme that confers resistance to H2O2. Microbiology 160, 47–55. 10.1099/mic.0.071365-0 24145018 PMC3917229

[B23] Glanemann C. Loos A. Gorret N. Willis L. B. O'Brien X. M. Lessard P. A.. (2003). Disparity between changes in mRNA abundance and enzyme activity in *Corynebacterium glutamicum*: implications for DNA microarray analysis. Appl. Microbiol. Biotechnol. 61, 61–68. 10.1007/s00253-002-1191-5 12658516

[B24] Gooday G. W. (1999). Aggressive and defensive roles for chitinases. EXS 87, 157–169. 10.1007/978-3-0348-8757-1_11 10906958

[B25] Hellberg A. Leippe M. Bruchhaus I. (2000). Two major 'higher molecular mass proteinases' of *Entamoeba histlytica* are identified as cysteine proteinases 1 and 2. Mol. Biochem. Parasitol. 105, 305–309. 10.1016/S0166-6851(99)00194-2 10693753

[B26] Hellberg A. Nickel R. Lotter H. Tannich E. Bruchhaus I. (2001). Overexpression of cysteine proteinase 2 in *Entamoeba histlytica* or *Entamoeba* dispar increases amoeba-induced monolayer destruction *in vitro* but does not augment amoebic liver abscess formation in gerbils. Cell. Microbiol. 3, 13–20. 10.1046/j.1462-5822.2001.00086.x 11207616

[B27] Heron B. T. Sateriale A. Teixeira J. E. Huston C. D. (2011). Evidence for a novel *Entamoeba histlytica* lectin activity that recognises carbohydrates present on ovalbumin. Int. J. Parasitol. 41, 137–144. 10.1016/j.ijpara.2010.07.011 20807536 PMC3003744

[B28] Herrero-Yraola A. Bakhit S. M. Franke P. Weise C. Schweiger M. Jorcke D.. (2001). Regulation of glutamate dehydrogenase by reversible ADP-ribosylation in mitochondria. EMBO J. 20, 2404–2412. 10.1093/emboj/20.10.2404 11350929 PMC125451

[B29] Husain A. Jeelani G. Sato D. Nozaki T. (2011). Global analysis of gene expression in response to L-cysteine deprivation in the anaerobic protozoan parasite *Entamoeba histlytica*. BMC Genomics 12:275. 10.1186/1471-2164-12-275 21627801 PMC3164229

[B30] Joseph N. Duppatla V. Rao D. N. (2006). Prokaryotic DNA mismatch repair. Prog. Nucleic Acid Res. Mol. Biol. 81, 1–49. 10.1016/S0079-6603(06)81001-9 16891168

[B31] Karpinets T. Greenwood D. Pogribny I. Samatova N. (2006). Bacterial stationary-state mutagenesis and Mammalian tumorigenesis as stress-induced cellular adaptations and the role of epigenetics. Curr. Genomics 7, 481–496. 10.2174/138920206779315764 18369407 PMC2269004

[B32] Kobe B. Kajava A. V. (2001). The leucine-rich repeat as a protein recognition motif. Curr. Opin. Struct. Biol. 11, 725–732. 10.1016/S0959-440X(01)00266-4 11751054

[B33] Koutsaimanis K. G. Timms P. W. Rée G. H. (1979). Failure of metronidazole in a patient with hepatic amebic abscess. Am. J. Trop. Med. Hyg. 28, 768–769. 464197

[B34] Kulda J. Cerkasov J. Demes P. Cerkasovová A. (1984). *Tritrichomonas foetus*: stable anaerobic resistance to metronidazole *in vitro*. Exp. Parasitol. 57, 93–103. 10.1016/0014-4894(84)90068-7 6692888

[B107] Kulda J. Kabíçková H. Tachezy J. Çerkasovová A. Çerkasov J. (1989). Metronidazole resistant trichomonads: mechanisms of in vitro developed anaerobic resistance, in Biochemistry and Molecular Biology of ‘Anaerobic’ Protozoa, eds Lloyd D. Coombs G. H. Paget T. A. P. (Chur: Harwood Academic Publishers), 137–160.

[B35] Lacey S. L. Moss S. F. Taylor G. W. (1993). Metronidazole uptake by sensitive and resistant isolates of *Helicobacter pylori*. J. Antimicrob. Chemother. 32, 393–400. 8262861 10.1093/jac/32.3.393

[B36] Ladner R. D. Lynch F. J. Groshen S. Xiong Y. P. Sherrod A. Caradonna S. J.. (2000). dUTP nucleotidohydrolase isoform expression in normal and neoplastic tissues: association with survival and response to 5-fluorouracil in colorectal cancer. Cancer Res. 60, 3493–3503. 10910061

[B37] Laity J. H. Lee B. M. Wright P. E. (2001). Zinc finger proteins: new insights into structural and functional diversity. Curr. Opin. Struct. Biol. 11, 39–46. 10.1016/S0959-440X(00)00167-6 11179890

[B38] Land K. M. Johnson P. J. (1999). Molecular basis of metronidazole resistance in pathogenic bacteria and protozoa. Drug Resist. Updat. 2, 289–294. 10.1054/drup.1999.0104 11504503

[B39] Lee K. W. Ko B. C. Jiang Z. Cao D. Chung S. S. (2001). Overexpression of aldose reductase in liver cancers may contribute to drug resistance. Anticancer Drugs 12, 129–132. 10.1097/00001813-200102000-00005 11261885

[B40] Lee W. P. (1993). The role of reduced growth rate in the development of drug resistance of HOB1 lymphoma cells to vincristine. Cancer Lett. 73, 105–111. 10.1016/0304-3835(93)90251-4 8221621

[B41] Leiros H. K. Kozielski-Stuhrmann S. Kapp U. Terradot L. Leonard G. A. McSweeney S. M. (2004). Structural basis of 5-nitroimidazole antibiotic resistance: the crystal structure of NimA from *Deinococcus radiodurans*. J. Biol. Chem. 279, 55840–55849. 10.1074/jbc.M408044200 15492014

[B42] Leitsch D. Burgess A. G. Dunn L. A. Krauer K. G. Tan K. Duchêne M.. (2011). Pyruvate:ferredoxin oxidoreductase and thioredoxin reductase are involved in 5-nitroimidazole activation while flavin metabolism is linked to 5-nitroimidazole resistance in *Giardia lamblia*. J. Antimicrob. Chemother. 66, 1756–1765. 10.1093/jac/dkr192 21602576 PMC3133484

[B43] Leitsch D. Kolarich D. Wilson I. B. Altmann F. Duchêne M. (2007). Nitroimidazole action in *Entamoeba histlytica*: a central role for thioredoxin reductase. PLoS Biol. 5:e211. 10.1371/journal.pbio.0050211 17676992 PMC1933457

[B44] Livak K. J. Schmittgen T. D. (2001). Analysis of relative gene expression data using real-time quantitative PCR and the 2(-Delta Delta C(T)) Method. Methods 25, 402–408. 10.1006/meth.2001.1262 11846609

[B45] Lo H. S. Reeves R. E. (1978). Pyruvate-to-ethanol pathway in *Entamoeba histlytica*. Biochem. J. 171, 225–230. 25658 10.1042/bj1710225 PMC1184151

[B46] Löfmark S. Edlund C. Nord C. E. (2010). Metronidazole is still the drug of choice for treatment of anaerobic infections. Clin. Infect. Dis. 50, S16–S23. 10.1086/647939 20067388

[B47] Loftus B. Anderson I. Davies R. Alsmark U. C. Samuelson J. Amedeo P.. (2005). The genome of the protist parasite *Entamoeba histolytica*. Nature 433, 865–868. 10.1038/nature03291 15729342

[B48] Ludlum D. B. Colinas R. J. Kirk M. C. Mehta J. R. (1988). Reaction of reduced metronidazole with guanosine to form an unstable adduct. Carcinogenesis 9, 593–596. 10.1093/carcin/9.4.593 3356067

[B49] Ma Q. Baldwin K. T. Renzelli A. J. McDaniel A. Dong L. (2001). TCDD-inducible poly(ADP-ribose) polymerase: a novel response to 2,3,7,8-tetrachlorodibenzo-p-dioxin. Biochem. Biophys. Res. Commun. 289, 499–506. 10.1006/bbrc.2001.5987 11716501

[B50] Marchler-Bauer A. Lu S. Anderson J. B. Chitsaz F. Derbyshire M. K. DeWeese-Scott C.. (2011). CDD: a Conserved Domain Database for the functional annotation of proteins. Nucleic Acids Res. 39, D225–D229. 10.1093/nar/gkq1189 21109532 PMC3013737

[B51] Marie C. Petri W. A. Jr. (2014). Regulation of virulence of *Entamoeba histolytica*. Annu. Rev. Microbiol. 68, 493–520. 10.1146/annurev-micro-091313-103550 25002094 PMC9006484

[B52] Marumo K. Nakada-Tsukui K. Tomii K. Nozaki T. (2014). Ligand heterogeneity of the cysteine protease binding protein family in the parasitic protist *Entamoeba histlytica*. Int. J. Parasitol. 44, 625–635. 10.1016/j.ijpara.2014.04.008 24907554

[B53] McLysaght A. Baldi P. F. Gaut B. S. (2003). Extensive gene gain associated with adaptive evolution of poxviruses. Proc. Natl. Acad. Sci. U.S.A. 100, 15655–15660. 10.1073/pnas.2136653100 14660798 PMC307623

[B54] Mirza H. Teo J. D. Upcroft J. Tan K. S. (2011a). A rapid, high-throughput viability assay for Blastocystis spp. *reveals metronidazole resistance and extensive subtype-dependent variations in drug susceptibilities*. Antimicrob. Agents Chemother. 55, 637–648. 10.1128/AAC.00900-10 21098237 PMC3028762

[B55] Mirza H. Wu Z. Kidwai F. Tan K. S. (2011b). A metronidazole-resistant isolate of Blastocystis spp. *is susceptible to nitric oxide and downregulates intestinal epithelial inducible nitric oxide synthase by a novel parasite survival mechanism*. Infect. Immun. 79, 5019–5026. 10.1128/IAI.05632-11 21930763 PMC3232666

[B56] Mishra V. Ali V. Nozaki T. Bhakuni V. (2010). *Entamoeba histlytica* Phosphoserine aminotransferase (EhPSAT): insights into the structure-function relationship. BMC Res. Notes 3:52. 10.1186/1756-0500-3-52 20199659 PMC2850911

[B57] Moreno S. N. Docampo R. (1985). Mechanism of toxicity of nitro compounds used in the chemotherapy of trichomoniasis. Environ. Health Perspect. 64, 199–208. 10.1289/ehp.8564199 3830698 PMC1568619

[B58] Müller J. Ley S. Felger I. Hemphill A. Müller N. (2008). Identification of differentially expressed genes in a *Giardia lamblia* WB C6 clone resistant to nitazoxanide and metronidazole. J. Antimicrob. Chemother. 62, 72–82. 10.1093/jac/dkn142 18408240

[B59] Müller J. Sterk M. Hemphill A. Müller N. (2007). Characterization of *Giardia lamblia* WB C6 clones resistant to nitazoxanide and to metronidazole. J. Antimicrob. Chemother. 60, 280–287. 10.1093/jac/dkm205 17561498

[B60] Müller M. Gorrell T. E. (1983). Metabolism and metronidazole uptake in *Trichomonas vaginalis* isolates with different metronidazole susceptibilities. Antimicrob. Agents Chemother. 24, 667–673. 10.1128/AAC.24.5.667 6607028 PMC185921

[B61] Müller M. Meingassner J. G. Miller W. A. Ledger W. J. (1980). Three metronidazole-resistant strains of *Trichomonas vaginalis* from the United States. Am. J. Obstet. Gynecol. 138, 808–812. 7004190 10.1016/s0002-9378(16)32741-7

[B62] Nakada-Tsukui K. Tsuboi K. Furukawa A. Yamada Y. Nozaki T. (2012). A novel class of cysteine protease receptors that mediate lysosomal transport. Cell. Microbiol. 14, 1299–1317. 10.1111/j.1462-5822.2012.01800.x 22486861 PMC3465781

[B63] Nakada-Tsukui K. Saito-Nakano Y. Ali V. Nozaki T. (2005). A retromerlike complex is a novel Rab7 effector that is involved in the transport of the virulence factor cysteine protease in the enteric protozoan parasite *Entamoeba histlytica*. Mol. Biol. Cell 16, 5294–5303. 10.1091/mbc.E05-04-0283 16120649 PMC1266427

[B64] Narcisi E. M. Secor W. E. (1996). *In vitro* effect of tinidazole and furazolidone on metronidazole-resistant *Trichomonas vaginalis*. Antimicrob. Agents Chemother. 40, 1121–1125. 8723451 10.1128/aac.40.5.1121 PMC163276

[B65] Nguewa P. A. Fuertes M. A. Cepeda V. Alonso C. Quevedo C. Soto M.. (2006). Poly(ADP-ribose) polymerase-1 inhibitor 3-aminobenzamide enhances apoptosis induction by platinum complexes in cisplatin-resistant tumor cells. Med. Chem. 2, 47–53. 10.2174/157340606775197697 16787355

[B66] Nozaki T. Ali V. Tokoro M. (2005). Sulfur-containing amino acid metabolism in parasitic protozoa. Adv. Parasitol. 60, 1–99. 10.1016/S0065-308X(05)60001-2 16230102

[B67] Pasupuleti V. Escobedo A. A. Deshpande A. Thota P. Roman Y. Hernandez A. V. (2014). Efficacy of 5-nitroimidazoles for the treatment of giardiasis: a systematic review of randomized controlled trials. PLoS Negl. Trop. Dis. 8:e2733. 10.1371/journal.pntd.0002733 24625554 PMC3953020

[B68] Pearson K. (1901). On lines and planes of closest fit to systems of points in space. Philos. Mag. 2, 559–572 10.1080/14786440109462720

[B69] Peláez T. Cercenado E. Alcalá L. Marín M. Martín-López A. Martínez-Alarcón J.. (2008). Metronidazole resistance in *Clostridium difficile* is heterogeneous. J. Clin. Microbiol. 46, 3028–3032. 10.1128/JCM.00524-08 18650353 PMC2546748

[B70] Penuliar G. M. Furukawa A. Nakada-Tsukui K. Husain A. Sato D. Nozaki T. (2012). Transcriptional and functional analysis of trifluoromethionine resistance in *Entamoeba histlytica*. J. Antimicrob. Chemother. 67, 375–386. 10.1093/jac/dkr484 22110087

[B71] Penuliar G. M. Furukawa A. Sato D. Nozaki T. (2011). Mechanism of trifluoromethionine resistance in *Entamoeba histlytica*. J. Antimicrob. Chemother. 66, 2045–2052. 10.1093/jac/dkr238 21676903

[B72] Plaitakis A. Zaganas I. (2001). Regulation of human glutamate dehydrogenases: implications for glutamate, ammonia and energy metabolism in brain. J. Neurosci. Res. 66, 899–908. 10.1002/jnr.10054 11746417

[B73] Pumbwe L. Chang A. Smith R. L. Wexler H. M. (2007). BmeRABC5 is a multidrug efflux system that can confer metronidazole resistance in *Bacteroides fragilis*. Microb. Drug Resist. 13, 96–101. 10.1089/mdr.2007.719 17650960

[B74] Quon D. V. d'Oliveira C. E. Johnson P. J. (1992). Reduced transcription of the ferredoxin gene in metronidazole-resistant *Trichomonas vaginalis*. Proc. Natl. Acad. Sci. U.S.A. 89, 4402–4406. 10.1073/pnas.89.10.4402 1374901 PMC49090

[B75] Ralph E. D. Clarke D. A. (1978). Inactivation of metronidazole by anaerobic and aerobic bacteria. Antimicrob. Agents Chemother. 14, 377–383. 10.1128/AAC.14.3.377 708015 PMC352468

[B108] Ralph E. D. Clarke J. T. Libke R. D. Luthy R. P. Kirby W. M. (1974). Pharmacokinetics of metronidazole as determined by bioassay. Antimicrob. Agents Chemother. 6, 691–696. 4451343 10.1128/aac.6.6.691 PMC444721

[B76] Ramana K. V. (2011). Aldose reductase: new insights for an old enzyme. Biomol. Concepts 2, 103–114. 10.1515/bmc.2011.002 21547010 PMC3085285

[B77] Rasoloson D. Vanácová S. Tomková E. Rázga J. Hrdy I. Tachezý J.. (2002). Mechanisms of *in vitro* development of resistance to metronidazole in *Trichomonas vaginalis*. Microbiology 148, 2467–2477. 12177340 10.1099/00221287-148-8-2467

[B78] Ravdin J. I. Murphy C. F. Salata R. A. Guerrant R. L. Hewlett E. L. (1985). N-Acetyl-D-galactosamine-inhibitable adherence lectin of *Entamoeba histlytica*. I. Partial purification and relation to amoebic virulence *in vitro*. J. Infect. Dis. 151, 804–815. 2859338 10.1093/infdis/151.5.804

[B79] Reuber T. L. Ausubel F. M. (1996). Isolation of Arabidopsis genes that differentiate between resistance responses mediated by the RPS2 and RPM1 disease resistance genes. Plant Cell 8, 241–249. 8742710 10.1105/tpc.8.2.241 PMC161094

[B80] Richards R. G. Sowers L. C. Laszlo J. Sedwick W. D. (1984). The occurrence and consequences of deoxyuridine in DNA. Adv. Enzyme Regul. 22, 157–185. 10.1016/0065-2571(84)90013-X 6147963

[B81] Samarawickrema N. A. Brown D. M. Upcroft J. A. Thammapalerd N. Upcroft P. (1997). Involvement of superoxide dismutase and pyruvate:ferredoxin oxidoreductase in mechanisms of metronidazole resistance in *Entamoeba histlytica*. J. Antimicrob. Chemother. 40, 833–840. 9462435 10.1093/jac/40.6.833

[B82] Samuelson J. (1999). Why metronidazole is active against both bacteria and parasites. Antimicrob. Agents Chemother. 43, 1533–1541. 10390199 10.1128/aac.43.7.1533 PMC89320

[B83] Sato D. Nakada-Tsukui K. Okada M. Nozaki T. (2006). Two cysteine protease inhibitors, EhICP1 and 2, localized in distinct compartments, negatively regulate secretion in *Entamoeba histlytica*. FEBS Lett. 580, 5306–5312. 10.1016/j.febslet.2006.08.081 16979632

[B84] Shain K. H. Dalton W. S. (2001). Cell adhesion is a key determinant in *de novo* multidrug resistance (MDR): new targets for the prevention of acquired MDR. Mol. Cancer Ther. 1, 69–78. 12467240

[B85] Sharma A. Sojar H. T. Glurich I. Honma K. Kuramitsu H. K. Genco R. J. (1998). Cloning, expression, and sequencing of a cell surface antigen containing a leucine-rich repeat motif from *Bacteroides forsythus* ATCC 43037. Infect. Immun. 66, 5703–5710. 9826345 10.1128/iai.66.12.5703-5710.1998 PMC108721

[B86] Sirinek K. R. (2000). Diagnosis and treatment of intra-abdominal abscesses. Surg. Infect. (Larchmt) 1, 31–38. 10.1089/109629600321272 12594907

[B87] Sisson G. Jeong J. Y. Goodwin A. Bryden L. Rossler N. Lim-Morrison S.. (2000). Metronidazole activation is mutagenic and causes DNA fragmentation in *Helicobacter pylori* and in *Escherichia coli* containing a cloned *H. pylori* RdxA(+) (Nitroreductase) gene. J. Bacteriol. 182, 5091–5096. 10.1128/JB.182.18.5091-5096.2000 10960092 PMC94656

[B88] Smith M. A. Edwards D. I. (1995). Redox potential and oxygen concentration as factors in the susceptibility of Helicobacter pylori to nitroheterocyclic drugs. J. Antimicrob. Chemother. 35, 751–764. 10.1093/jac/35.6.751 7559187

[B89] Stigler S. M. (1989). Francis galton's account of the invention of correlation. Stat. Sci. 4, 73–79 10.1214/ss/1177012580

[B90] Tabatabaie L. Klomp L. W. Berger R. de Koning T. J. (2010). L-serine synthesis in the central nervous system: a review on serine deficiency disorders. Mol. Genet. Metab. 99, 256–262. 10.1016/j.ymgme.2009.10.012 19963421

[B91] Tanih N. F. Ndip L. M. Ndip R. N. (2011). Characterisation of the genes encoding resistance to metronidazole (rdxA and frxA) and clarithromycin (the 23S-rRNA genes) in South African isolates of *Helicobacter pylori*. Ann. Trop. Med. Parasitol. 105, 251–259. 10.1179/136485911X12899838683485 21801504 PMC4090784

[B92] Tazreiter M. Leitsch D. Hatzenbichler E. Mair-Scorpio G. E. Steinborn R. Schreiber M.. (2008). *Entamoeba histlytica*: response of the parasite to metronidazole challenge on the levels of mRNA and protein expression. Exp. Parasitol. 120, 403–410. 10.1016/j.exppara.2008.09.011 18845147

[B93] Tejman-Yarden N. Millman M. Lauwaet T. Davids B. J. Gillin F. D. Dunn L.. (2011). Impaired parasite attachment as fitness cost of metronidazole resistance in *Giardia lamblia*. Antimicrob. Agents Chemother. 55, 4643–4651. 10.1128/AAC.00384-11 21825286 PMC3186953

[B94] Tillack M. Biller L. Irmer H. Freitas M. Gomes M. A. Tannich E.. (2007). The *Entamoeba histolytica* genome: primary structure and expression of proteolytic enzymes. BMC Genomics 8:170. 10.1186/1471-2164-8-170 17567921 PMC1913524

[B95] Townson S. M. Laqua H. Upcroft P. Boreham P. F. Upcroft J. A. (1992). Induction of metronidazole and furazolidone resistance in *Giardia*. Trans. R. Soc. Trop. Med. Hyg. 86, 521–522. 10.1016/0035-9203(92)90095-T 1475822

[B96] Tukey J. W. (1949). Comparing individual means in the analysis of variance. Biometrics 5, 99–114. 10.2307/3001913 18151955

[B97] Upcroft J. A. Upcroft P. (1993). Drug resistance and *Giardia*. Parasitol. Today 9, 187–190. 10.1016/0169-4758(93)90144-5 15463750

[B98] Van Oosten M. A. Notten F. J. Mikx F. H. (1986). Metronidazole concentrations in human plasma, saliva, and gingival crevice fluid after a single dose. J. Dent. Res. 65, 1420–1423. 10.1177/00220345860650120801 3097094

[B99] Vicente J. B. Ehrenkaufer G. M. Saraiva L. M. Teixeira M. Singh U. (2009). *Entamoeba histlytica* modulates a complex repertoire of novel genes in response to oxidative and nitrosative stresses: implications for amebic pathogenesis. Cell Microbiol. 11, 51–69. 10.1111/j.1462-5822.2008.01236.x 18778413 PMC3418052

[B100] Wassmann C. Hellberg A. Tannich E. Bruchhaus I. (1999). Metronidazole resistance in the protozoan parasite *Entamoeba histlytica* is associated with increased expression of iron-containing superoxide dismutase and peroxiredoxin and decreased expression of ferredoxin 1 and flavin reductase. J. Biol. Chem. 274, 26051–26056. 10.1074/jbc.274.37.26051 10473552

[B101] Welch B. L. (1947). The generalization of “Student's” problem when several different population variances are involved. Biometrika 34, 28–35. 10.1093/biomet/34.1-2.28 20287819

[B102] West S. B. Wislocki P. G. Fiorentini K. M. Alvaro R. Wolf F. J. Lu A. Y. (1982). Drug residue formation from ronidazole, a 5-nitroimidazole. *I. Characterization of in vitro protein alkylation*. Chem. Biol. Interact. 41, 265–279. 10.1016/0009-2797(82)90105-3 6809345

[B103] World Health Organization Amoebiasis. (1997). WHO/PAHO/UNESCO report. A consultation with experts on amoebiasis. Mexico City, Mexico 28-29 January, 1997. Epidemiol. Bull. 18, 13–4. 9197085

[B104] Wright J. M. Webb R. I. O'Donoghue P. Upcroft P. Upcroft J. A. (2010). Hydrogenosomes of laboratory-induced metronidazole-resistant *Trichomonas vaginalis* lines are downsized while those from clinically metronidazole-resistant isolates are not. J. Eukaryot. Microbiol. 57, 171–176. 10.1111/j.1550-7408.2009.00455.x 20015182

[B105] Yarlett N. Yarlett N. C. Lloyd D. (1986). Ferredoxin-dependent reduction of nitroimidazole derivatives in drug-resistant and susceptible strains of *Trichomonas vaginalis*. Biochem. Pharmacol. 35, 1703–1708. 10.1016/0006-2952(86)90327-8 3486660

[B106] Yoshikawa T. T. Miyamoto S. Chow A. W. Guze L. B. (1974). *In vitro* resistance of *Neisseria gonorrhoeae* to metronidazole. Antimicrob. Agents Chemother. 6, 327–329. 10.1128/AAC.6.3.327 15830482 PMC444646

