# Supplementary material for: Phenotypic and transcriptional profiling in Entamoeba histolytica reveal costs to fitness and adaptive responses associated with metronidazole resistance
Source: Front Microbiol. 2015 May 5;6:354. doi: 10.3389/fmicb.2015.00354 (PMC4419850; doi:10.3389/fmicb.2015.00354)
Supplement: Supplementary file 1 [file Table1.DOC]

Table S1. Primers used in qRT-PCR.

| Primer | Sequence (5’🡪3’) |
| --- | --- |
| Acetyltransferase.F | TGAAGACGGTGCATGGATTGGGT |
| Acetyltransferase.R | CTTGCAGGATTTCCTACAGCAACCA |
| AIG1-A.F | ACTCCCATCCAGATGAAGGGTGTGA |
| AIG1-A.R | TCAGCTTCTACTCGGCGCTGT |
| AIG1-B.F | AGTTGGTGGTGGTTCAGCATCAGT |
| AIG1-B.R | TCAACCACGCTTTGTGGTGTGT |
| DNApol.F | TGGTGGAAGGCGGCGACGAT |
| DNApol.R | TAAGTACACCGGACTGATGGAGTGC |
| HP1.F | TGGGAGTAGTGAAGCAGCAGTCA |
| HP1.R | ACAATCGGTTGGGTTGTTAACACGA |
| HP2.F | TGAGCACTTTTAAAACCCACCGTGA |
| HP2.R | ACTGGGCGTTGAATAAGAAACGAGT |
| HP3.F | TGTGGGCTAAGTGGGACCGCA |
| HP3.R | CCACCCACCTCTTCCCAGGGT |
| HP4.F | CCAGGAGCACCAGCAACCGT |
| HP4.R | GCTGATTCTCCTCCAGCGTGTGT |
| Hydrolase.F | ACTTACTACTCTTCCCGCTGAGCA |
| Hydrolase.R | AGCAGCAACAATAGCTTCATCAGCA |
| ISF1.F | GTTGGCAAAAGGATCCTGTGATGGA |
| ISF1.R | CTTGCCATAACAACTGCTGCTCCT |
| ISF2.F | GGATGTGCTGGATGTCTTGCATGT |
| ISF2.R | CCTCGATCATAACCAGCAGAACCAC |
| ISF-A.F | TGTTGGAGACGCAACACCTCCT |
| ISF-A.R | TCACACCTTCCCCACTCCATCCA |
| NADglutamate.F | GGTGGACCAAATGGTGATGTTGCT |
| NADglutamate.R | TGGCCTACCACCACATGGACA |
| Phosphatase.F | ACTTGGGCATATTCACCGTGATGAG |
| Phosphatase.R | AGTTCTCCATCGGTACAATGACCCT |
| TK.F | TGTCAATGGAGTTTGTGGTGGAACA |
| TK.R | GGCATCCAGAAAGACATGGAGAACA |
