# Supplementary material for: Phenotypic and transcriptional profiling in Entamoeba histolytica reveal costs to fitness and adaptive responses associated with metronidazole resistance
Source: Front Microbiol. 2015 May 5;6:354. doi: 10.3389/fmicb.2015.00354 (PMC4419850; doi:10.3389/fmicb.2015.00354)
Supplement: Supplementary file 2 [file Table2.DOC]

Table S2. Primers used to construct plasmids for protein overexpression.

| Primer | Sequence (5’- 3’) | Product size (bp) |
| --- | --- | --- |
| HA.HP.ZFP.F | TCCCCCGGGATGACTAACCAACTCATCAAAGAACTG | 504 |
| HA.HP.ZFP.R | CCGCTCGAGTCAAAGTGGTAATTGTGGTGGA |
| HA.ISF1.F | TCCCCCGGGATGACAAAACAAATTAAGGTATTATTGATT | 588 |
| HA.ISF1.R | CCGCTCGAGTTACTCAACTATTCTCTTAAGAACAAATGC |
| HA.ISF2.F | TCCCCCGGGATGTCTTTAAAAGTTCTTACACTTCTTGC | 579 |
| HA.ISF2.R | CCGCTCGAGTTAAATTTTTTGTGCAATTTTTTCTCC |
| HA.ISF-A.F | TCCCCCGGGATGCAGAATTTAGCTGTTAAAGTAGTAGAT | 1236 |
| HA.ISF-A.R | CCGCTCGAGTTAATGATTATGATCTATTACTTTTGATCC |
| HA.ISF-B.F | TCCCCCGGGATGGTTGCTAAAGTTCTTGTTCTTCT | 606 |
| HA.ISF-B.R | CCGCTCGAGTTAAAATTGATTGTTCTGTTTCATTAATTTCTC |

Nucleotide overhangs are marked by single underlining, restriction sites for SmaI by double underlining, and restriction sites for XhoI by dotted underlining.
