# Supplementary material for: Phenotypic and transcriptional profiling in Entamoeba histolytica reveal costs to fitness and adaptive responses associated with metronidazole resistance
Source: Front Microbiol. 2015 May 5;6:354. doi: 10.3389/fmicb.2015.00354 (PMC4419850; doi:10.3389/fmicb.2015.00354)
Supplement: Supplementary file 4 [file Table4.DOC]

Table S4. List of hypothetical proteins upregulated by >3 fold in MTZR (+).

| Probe Set ID | NCBI RefSeq | p-value | Fold change | Regulation |
| --- | --- | --- | --- | --- |
| EHI_006850_at | XM_649369 | 1.72E-04 | 36.9 | upregulated |
| 72.m00179_at | XM_648717 | 1.78E-03 | 30.9 | downregulated |
| EHI_165190_at | XM_647137 | 1.45E-03 | 27.1 | upregulated |
| EHI_127670_at | XM_644313 | 2.14E-04 | 17.0 | downregulated |
| EHI_144150_s_at | XM_001914451 | 5.39E-03 | 16.2 | upregulated |
| 194.m00101_s_at | XM_645779 | 1.03E-03 | 12.1 | upregulated |
| EHI_159810_s_at | XM_643404 | 4.86E-03 | 11.7 | downregulated |
| EHI_002240_s_at | XM_645752 | 7.44E-03 | 11.4 | upregulated |
| EHI_196720_s_at | XM_643106 | 2.53E-02 | 10.9 | upregulated |
| EHI_118420_at | XM_001913557 | 4.42E-03 | 9.5 | upregulated |
| EHI_050490_at | XM_651482 | 5.98E-03 | 9.1 | upregulated |
| EHI_183210_s_at | XM_650406 | 4.44E-03 | 9.1 | upregulated |
| 375.m00058_s_at | XM_643788 | 6.92E-03 | 9.1 | upregulated |
| EHI_087210_at | XM_643131 | 8.21E-03 | 8.9 | upregulated |
| EHI_136840_s_at | XM_001914556 | 1.29E-04 | 7.3 | upregulated |
| EHI_014910_s_at | XM_001914428 | 2.83E-02 | 7.2 | upregulated |
| EHI_147860_at | XM_642848 | 7.90E-03 | 7.2 | downregulated |
| EHI_029500_s_at | XM_644980 | 3.80E-03 | 6.8 | upregulated |
| EHI_067250_at | XM_647565 | 1.89E-02 | 6.7 | downregulated |
| EHI_011560_s_at | XM_648116 | 4.82E-05 | 6.7 | upregulated |
| EHI_166690_at | XM_001913536 | 5.56E-03 | 6.5 | upregulated |
| 363.m00056_s_at | XM_643862 | 3.73E-03 | 6.3 | upregulated |
| EHI_129830_at | XM_646717 | 3.85E-03 | 6.2 | downregulated |
| 397.m00061_s_at | XM_643667 | 6.65E-04 | 6.2 | upregulated |
| EHI_059280_s_at | XM_001914072 | 3.21E-04 | 6.0 | upregulated |
| EHI_037700_s_at | XM_643865 | 9.75E-03 | 6.0 | upregulated |
| EHI_162780_s_at | XM_650359 | 6.75E-03 | 6.0 | upregulated |
| EHI_091350_s_at | XM_001914397 | 3.41E-02 | 5.9 | upregulated |
| EHI_109250_s_at | XM_001913927 | 4.67E-02 | 5.9 | downregulated |
| EHI_018270_s_at | XM_001914263 | 1.68E-03 | 5.9 | upregulated |
| 190.m00086_s_at | XM_645857 | 6.39E-03 | 5.8 | upregulated |
| EHI_045820_s_at | XM_001914519 | 1.02E-03 | 5.7 | upregulated |
| 522.m00019_s_at | XM_643098 | 1.90E-02 | 5.7 | upregulated |
| EHI_047630_s_at | XM_001914393 | 1.63E-02 | 5.7 | downregulated |
| 50.m00196_s_at | XM_649450 | 6.51E-03 | 5.7 | upregulated |
| 654.m00031_s_at | XM_642894 | 8.24E-05 | 5.6 | upregulated |
| EHI_074520_s_at | XM_645865 | 1.01E-02 | 5.5 | upregulated |
| 462.m00042_s_at | XM_643313 | 8.76E-04 | 5.4 | upregulated |
| EHI_191730_at | XM_643923 | 4.85E-03 | 5.4 | upregulated |
| EHI_187080_at | XM_651386 | 1.53E-03 | 5.1 | downregulated |
| 266.m00066_s_at | XM_644755 | 2.52E-02 | 5.1 | upregulated |
| 506.m00025_s_at | XM_643137 | 1.04E-03 | 5.1 | upregulated |
| EHI_034530_s_at | XM_643791 | 2.99E-03 | 5.1 | upregulated |
| EHI_164170_s_at | XM_001913664 | 2.56E-02 | 4.9 | upregulated |
| EHI_054680_at | XM_646972 | 3.36E-03 | 4.8 | downregulated |
| EHI_141050_at | XM_001913802 | 5.02E-03 | 4.7 | upregulated |
| EHI_034590_s_at | XM_001914026 | 2.72E-03 | 4.6 | upregulated |
| EHI_010130_at | XM_651999 | 7.41E-03 | 4.4 | upregulated |
| EHI_067260_at | XM_647564 | 4.12E-04 | 4.3 | downregulated |
| EHI_165450_at | XM_642785 | 7.23E-03 | 4.3 | upregulated |
| EHI_100250_at | XM_646618 | 4.90E-03 | 4.2 | upregulated |
| EHI_114950_at | XM_645239 | 3.50E-02 | 4.2 | downregulated |
| EHI_167450_s_at | XM_001914217 | 1.71E-02 | 4.1 | upregulated |
| EHI_141030_at | XM_649510 | 1.73E-04 | 4.1 | upregulated |
| 363.m00048_at | XM_643868 | 1.88E-02 | 4.0 | downregulated |
| EHI_165200_at | XM_647138 | 3.40E-03 | 4.0 | upregulated |
| EHI_062960_at | XM_645139 | 4.41E-03 | 4.0 | downregulated |
| 248.m00060_s_at | XM_644979 | 4.90E-02 | 3.9 | upregulated |
| EHI_075710_at | XM_643683 | 2.25E-02 | 3.9 | upregulated |
| EHI_004520_at | XM_651631 | 5.71E-03 | 3.8 | upregulated |
| 22.m00285_at | XM_650728 | 4.56E-02 | 3.8 | downregulated |
| 86.m00176_at | XM_648229 | 4.33E-02 | 3.7 | upregulated |
| EHI_073520_at | XM_001913502 | 6.67E-03 | 3.7 | downregulated |
| 214.m00066_s_at | XM_645422 | 9.42E-03 | 3.6 | upregulated |
| 397.m00056_s_at | XM_643660 | 2.02E-02 | 3.5 | upregulated |
| 77.m00173_at | XM_648536 | 8.56E-04 | 3.4 | downregulated |
| EHI_137240_at | XM_648760 | 3.01E-02 | 3.4 | downregulated |
| 458.m00053_at | XM_643337 | 2.59E-03 | 3.4 | upregulated |
| EHI_089000_s_at | XM_001914320 | 2.70E-02 | 3.4 | downregulated |
| EHI_114650_at | XM_645241 | 4.15E-02 | 3.3 | upregulated |
| EHI_058480_at | XM_646296 | 4.25E-02 | 3.3 | downregulated |
| EHI_056820_at | XM_642890 | 6.20E-03 | 3.3 | downregulated |
| EHI_054700_at | XM_646974 | 1.94E-03 | 3.3 | downregulated |
| EHI_032670_s_at | XM_645799 | 1.39E-03 | 3.3 | upregulated |
| EHI_069940_at | XM_643688 | 2.94E-02 | 3.3 | downregulated |
| EHI_174600_at | XM_001913588 | 1.14E-02 | 3.2 | upregulated |
| EHI_156680_at | XM_645069 | 3.94E-02 | 3.2 | upregulated |
| EHI_095480_at | XM_644124 | 1.17E-02 | 3.2 | upregulated |
| EHI_012990_at | XM_651227 | 2.97E-02 | 3.2 | upregulated |
| 283.m00056_at | XM_644538 | 7.41E-03 | 3.2 | upregulated |
| EHI_152200_at | XM_650709 | 2.09E-02 | 3.2 | downregulated |
| EHI_129880_at | XM_646722 | 4.94E-03 | 3.1 | upregulated |
| EHI_067600_at | XM_652057 | 4.82E-03 | 3.1 | downregulated |
| EHI_027030_at | XM_643663 | 1.62E-02 | 3.0 | upregulated |
| 72.m00186_at | XM_648694 | 2.16E-03 | 3.0 | downregulated |
| EHI_023150_at | XM_650565 | 4.64E-03 | 3.0 | upregulated |
| EHI_049960_at | XM_651359 | 1.05E-03 | 3.0 | downregulated |
