# Supplementary material for: Phenotypic and transcriptional profiling in Entamoeba histolytica reveal costs to fitness and adaptive responses associated with metronidazole resistance
Source: Front Microbiol. 2015 May 5;6:354. doi: 10.3389/fmicb.2015.00354 (PMC4419850; doi:10.3389/fmicb.2015.00354)
Supplement: Supplementary file 5 [file Table5.DOC]

Table S5. List of all GO terms associated with genes modulated in MTZR.

| Probe Set ID | GO IDs | | | | | |
| --- | --- | --- | --- | --- | --- | --- |
| EHI_033560_s_at | GO:0008150 | GO:0005886 | GO:0003674 |  |  |  |
| EHI_077280_s_at | GO:0008150 | GO:0003674 |  |  |  |  |
| 371.m00031_s_at | GO:0008150 | GO:0005886 | GO:0003674 |  |  |  |
| EHI_164190_at | GO:0006260 | GO:0000166 | GO:0003676 | GO:0003677 | GO:0003887 | GO:0008408 |
| EHI_025710_at | GO:0003955 | GO:0009055 | GO:0016491 | GO:0050662 | GO:0006118 |  |
| 522.m00018_at | GO:0005525 |  |  |  |  |  |
| 628.m00011_at | GO:0005525 |  |  |  |  |  |
| EHI_160330_s_at | GO:0006508 | GO:0008234 | GO:0004215 |  |  |  |
| EHI_121160_s_at | GO:0006508 | GO:0008234 | GO:0004215 |  |  |  |
| EHI_189960_at | GO:0006886 | GO:0007264 | GO:0045184 | GO:0005622 | GO:0005525 | GO:0030701 |
| EHI_011560_s_at | GO:0006508 |  |  |  |  |  |
| EHI_026000_s_at | GO:0005525 | GO:0008234 |  |  |  |  |
| EHI_138480_at | GO:0008752 | GO:0006118 |  |  |  |  |
| 522.m00019_s_at | GO:0005525 |  |  |  |  |  |
| EHI_129890_at | GO:0009055 | GO:0010181 | GO:0016491 | GO:0016787 | GO:0046872 |  |
| EHI_022600_s_at | GO:0008752 | GO:0006118 |  |  |  |  |
| EHI_181710_s_at | GO:0008752 | GO:0006118 |  |  |  |  |
| EHI_096770_at | GO:0016740 |  |  |  |  |  |
| EHI_067720_s_at | GO:0008752 | GO:0006118 |  |  |  |  |
| EHI_103260_s_at | GO:0008752 | GO:0006118 |  |  |  |  |
| EHI_074750_at | GO:0007264 | GO:0015031 | GO:0005622 | GO:0004767 | GO:0005525 |  |
| EHI_148550_at | GO:0006468 | GO:0004672 | GO:0004674 | GO:0004713 | GO:0005524 |  |
| EHI_075660_at | GO:0006508 | GO:0016020 | GO:0004222 |  |  |  |
| 214.m00066_s_at | GO:0006508 | GO:0008234 |  |  |  |  |
| EHI_075150_at | GO:0006520 | GO:0008152 | GO:0055114 | GO:0003824 | GO:0005488 | GO:0016491 |
| EHI_179060_at | GO:0003976 |  |  |  |  |  |
| EHI_126550_at | GO:0005525 |  |  |  |  |  |
| EHI_082060_at | GO:0008150 | GO:0003674 |  |  |  |  |
| EHI_020250_at | GO:0006629 | GO:0008150 | GO:0003674 | GO:0004607 |  |  |
| 36.m00218_s_at | GO:0005524 | GO:0008559 | GO:0016887 |  |  |  |
| 2.m00624_s_at | GO:0006468 | GO:0004672 | GO:0004674 | GO:0004713 | GO:0005524 |  |
| EHI_147020_at | GO:0051056 | GO:0005622 | GO:0004767 | GO:0005096 | GO:0016787 |  |
| EHI_006140_at | GO:0035023 | GO:0005622 | GO:0005089 |  |  |  |
| EHI_026360_s_at | GO:0006564 | GO:0004648 | GO:0008483 |  |  |  |
| EHI_045450_at | GO:0007264 | GO:0015031 | GO:0005622 | GO:0005525 |  |  |
| EHI_029620_s_at | GO:0055114 | GO:0004032 | GO:0016491 |  |  |  |
| EHI_118410_at | GO:0006468 | GO:0004672 | GO:0004674 | GO:0004713 | GO:0005524 |  |
| EHI_075640_at | GO:0003824 | GO:0004721 |  |  |  |  |
| EHI_045600_at | GO:0007264 | GO:0008150 | GO:0005622 | GO:0003674 | GO:0005525 |  |
| EHI_067220_at | GO:0006886 | GO:0006913 | GO:0007165 | GO:0007264 | GO:0015031 | GO:0005622 |
|  | GO:0003924 | GO:0005515 | GO:0005525 |  |  |  |
| EHI_061760_at | GO:0005488 |  |  |  |  |  |
| EHI_091450_at | GO:0006508 | GO:0008234 | GO:0004215 |  |  |  |
