# Supplementary material for: Phenotypic and transcriptional profiling in Entamoeba histolytica reveal costs to fitness and adaptive responses associated with metronidazole resistance
Source: Front Microbiol. 2015 May 5;6:354. doi: 10.3389/fmicb.2015.00354 (PMC4419850; doi:10.3389/fmicb.2015.00354)
Supplement: Supplementary file 6 [file Table6.DOC]

Table S6. Transmembrane domain and signal peptide predictions of the genes differentially expressed in MTZR.

| Probe set ID | Transmembrane domain prediction | | | Signal peptide prediction | | |
| --- | --- | --- | --- | --- | --- | --- |
|  | SOSUI | TMHMM2 | Phobius | Phobius | SignalP | PrediSi |
| 190.m00086_s_at | 1 | 1 | 1 | none | none | none |
| 194.m00101_s_at | none | None | none | none | none | none |
| 2.m00624_s_at | 1 | 2 | 2 | none | none | none |
| 214.m00066_s_at | 1 | None | none | none | none | none |
| 22.m00285_at | 1 | 1 | none | present | present | none |
| 248.m00060_s_at | none | None | none | none | none | none |
| 266.m00066_s_at | none | None | none | none | none | none |
| 283.m00056_at | none | None | none | none | none | none |
| 36.m00218_s_at | none | None | none | none | none | none |
| 363.m00048_at | none | None | none | none | none | none |
| 363.m00056_s_at | none | None | none | none | none | none |
| 371.m00031_s_at | none | None | none | none | none | none |
| 375.m00058_s_at | none | None | none | none | none | none |
| 397.m00056_s_at | none | None | none | none | none | none |
| 397.m00061_s_at | none | none | none | none | none | none |
| 432.m00028_at | none | none | none | none | none | none |
| 458.m00053_at | none | none | none | none | none | none |
| 462.m00042_s_at | none | none | none | none | none | none |
| 50.m00196_s_at | none | none | none | none | none | none |
| 506.m00025_s_at | 1 | 1 | 1 | none | none | none |
| 522.m00018_at | none | none | none | none | none | none |
| 522.m00019_s_at | none | none | none | none | none | none |
| 554.m00020_s_at | none | none | none | none | none | none |
| 628.m00011_at | 2 | 3 | 3 | none | none | none |
| 654.m00031_s_at | none | none | none | none | none | none |
| 72.m00179_at | 2 | 2 | 2 | none | none | none |
| 72.m00186_at | none | none | none | none | none | none |
| 77.m00173_at | none | none | none | none | none | none |
| 82.m00157_s_at | none | none | none | present | present | none |
| 82.m00164_s_at | none | none | none | present | present | none |
| 86.m00176_at | none | none | none | none | none | none |
| EHI_002240_s_at | none | none | none | none | none | none |
| EHI_004520_at | none | none | none | none | none | none |
| EHI_006140_at | none | none | none | none | none | none |
| EHI_006850_at | none | none | none | none | none | none |
| EHI_010130_at | none | none | none | none | none | none |
| EHI_011560_s_at | 2 | none | 1 | present | present | none |
| EHI_012990_at | none | none | none | none | none | none |
| EHI_014910_s_at | none | none | none | none | none | none |
| EHI_018270_s_at | none | none | none | none | none | none |
| EHI_020250_at | none | none | none | present | present | present |
| EHI_022270_s_at | none | none | none | present | present | none |
| EHI_022600_s_at | none | none | none | present | present | none |
| EHI_023150_at | none | none | none | none | none | none |
| EHI_025710_at | none | none | none | none | none | none |
| EHI_026000_s_at | none | none | 2 | none | none | none |
| EHI_026360_s_at | none | none | none | none | none | none |
| EHI_027030_at | none | none | none | none | none | none |
| EHI_029500_s_at | none | none | none | none | none | none |
| EHI_029620_s_at | none | none | none | none | none | none |
| EHI_032670_s_at | 2 | none | none | none | none | none |
| EHI_033560_s_at | none | none | none | none | none | none |
| EHI_034530_s_at | none | none | none | none | none | none |
| EHI_034590_s_at | none | none | none | none | none | none |
| EHI_037700_s_at | none | none | none | none | none | none |
| EHI_045450_at | none | none | none | none | none | none |
| EHI_045600_at | none | none | none | none | none | none |
| EHI_045820_s_at | none | none | none | none | none | none |
| EHI_047630_s_at | none | none | none | present | present | none |
| EHI_049960_at | none | none | none | none | none | none |
| EHI_050490_at | none | none | 1 | none | none | none |
| EHI_054680_at | none | none | none | present | none | none |
| EHI_054690_at | none | none | none | none | none | none |
| EHI_054700_at | none | none | none | none | none | none |
| EHI_056820_at | none | none | none | none | none | none |
| EHI_058480_at | none | none | none | none | none | none |
| EHI_059280_s_at | none | none | none | none | none | none |
| EHI_061760_at | none | none | none | none | none | none |
| EHI_062960_at | none | 1 | 1 | present | present | none |
| EHI_067220_at | none | none | none | none | none | none |
| EHI_067250_at | none | none | none | present | none | none |
| EHI_067260_at | none | none | none | none | none | none |
| EHI_067600_at | 2 | 1 | 1 | present | present | present |
| EHI_067720_s_at | none | none | none | present | present | none |
| EHI_069940_at | none | none | none | none | none | none |
| EHI_072000_s_at | none | none | 1 | present | present | present |
| EHI_072960_s_at | none | none | none | none | none | none |
| EHI_073520_at | 1 | 1 | 1 | none | none | present |
| EHI_073980_s_at | none | none | 1 | present | present | present |
| EHI_074520_s_at | none | none | none | none | none | none |
| EHI_074750_at | none | none | none | none | none | none |
| EHI_075150_at | none | none | none | none | none | present |
| EHI_075640_at | 1 | none | none | none | none | none |
| EHI_075660_at | 7 | 7 | 7 | none | none | none |
| EHI_075710_at | none | none | none | none | none | none |
| EHI_077280_s_at | none | none | none | none | none | none |
| EHI_082060_at | none | none | none | none | none | none |
| EHI_087210_at | 1 | none | 1 | present | present | present |
| EHI_089000_s_at | none | none | none | present | present | none |
| EHI_090260_at | none | none | none | none | none | none |
| EHI_091350_s_at | 1 | none | none | none | none | none |
| EHI_091450_at | 2 | 1 | 1 | present | none | none |
| EHI_092100_at | 1 | none | none | present | present | none |
| EHI_095480_at | 3 | 3 | 5 | none | none | present |
| EHI_096770_at | none | none | none | none | none | none |
| EHI_100250_at | none | none | none | none | none | none |
| EHI_103260_s_at | none | none | none | present | present | none |
| EHI_109250_s_at | none | none | none | present | present | none |
| EHI_114650_at | none | none | none | none | none | none |
| EHI_114950_at | none | none | none | none | none | none |
| EHI_118410_at | 2 | 1 | 1 | present | present | present |
| EHI_118420_at | none | none | none | none | none | none |
| EHI_121160_s_at | none | none | none | present | present | present |
| EHI_126550_at | none | none | none | none | none | none |
| EHI_126560_at | none | none | none | none | none | none |
| EHI_127670_at | none | none | none | none | none | none |
| EHI_129830_at | none | none | none | none | none | none |
| EHI_129880_at | none | none | none | none | none | none |
| EHI_129890_at | none | none | none | none | none | none |
| EHI_136840_s_at | none | none | none | none | none | none |
| EHI_137240_at | none | none | none | none | none | none |
| EHI_138480_at | none | none | none | none | none | none |
| EHI_141030_at | none | none | none | none | none | none |
| EHI_141050_at | none | none | none | none | none | none |
| EHI_144150_s_at | none | none | none | none | none | none |
| EHI_147020_at | none | none | none | present | present | none |
| EHI_147860_at | none | 1 | 1 | none | none | none |
| EHI_148550_at | 1 | 2 | 1 | none | none | none |
| EHI_152200_at | none | none | none | none | none | none |
| EHI_156680_at | none | none | none | none | none | none |
| EHI_159810_s_at | none | none | none | present | present | none |
| EHI_160330_s_at | none | none | 1 | none | none | none |
| EHI_162780_s_at | none | none | none | none | none | none |
| EHI_164170_s_at | none | none | none | none | none | none |
| EHI_164190_at | none | none | none | none | none | none |
| EHI_165190_at | none | none | none | none | none | none |
| EHI_165200_at | none | none | none | none | none | none |
| EHI_165450_at | none | none | none | none | none | none |
| EHI_166690_at | 1 | 1 | 1 | none | none | none |
| EHI_167450_s_at | none | none | none | none | none | none |
| EHI_174230_s_at | none | none | none | none | none | none |
| EHI_174600_at | none | none | none | none | none | none |
| EHI_176580_at | none | none | none | none | none | none |
| EHI_176700_at | none | none | none | none | none | none |
| EHI_179060_at | 1 | 1 | 1 | none | none | none |
| EHI_180390_at | none | 1 | 1 | none | none | none |
| EHI_181710_s_at | none | none | none | present | present | none |
| EHI_183210_s_at | 2 | none | none | present | present | none |
| EHI_187080_at | none | none | none | present | present | present |
| EHI_189960_at | none | none | None | none | none | none |
| EHI_191730_at | 2 | 1 | 1 | present | present | Present |
| EHI_196720_s_at | none | none | None | none | none | None |
