# Supplementary material for: Phenotypic and transcriptional profiling in Entamoeba histolytica reveal costs to fitness and adaptive responses associated with metronidazole resistance
Source: Front Microbiol. 2015 May 5;6:354. doi: 10.3389/fmicb.2015.00354 (PMC4419850; doi:10.3389/fmicb.2015.00354)
Supplement: Supplementary file 7 [file Table7.DOC]

Table S7. DNA molecule location of genes

| Probe Set ID | Fold change | Regulation | Molecule |
| --- | --- | --- | --- |
| EHI_089000_s_at | 3.4 | Downregulated | 1101698136023 6881 bp 4 c |
| EHI_181710_s_at | 5.3 | Upregulated | 1101698136024 1737 bp 1 c |
| EHI_027030_at | 3 | Upregulated | 1101698136058 6710 bp 3 c |
| EHI_056820_at | 3.3 | Downregulated | 1101698136094 2521 bp 1 c |
| EHI_061760_at | 3 | Downregulated | 1101698136111 17046 bp 4 |
| EHI_045820_s_at | 5.7 | Upregulated | 1101698136120 1692 bp 1 c |
| EHI_014910_s_at | 7.2 | Upregulated | 1101698136148 3148 bp 1 c |
| EHI_121160_s_at | 7.3 | Downregulated | 1101698136168 3323 bp 1 c |
| EHI_147020_at | 3.3 | Upregulated | 1101698136260 25830 bp 2 |
| EHI_144150_s_at | 16.2 | Upregulated | 1101698136264 2624 bp 1 c |
| EHI_165450_at | 4.3 | Upregulated | 1101698136280 4404 bp 2 c |
| EHI_072960_s_at | 4.9 | Upregulated | 1101698136311 1752 bp 1 c |
| EHI_126550_at | 3.5 | Upregulated | 1101698136470 13765 bp 1 |
| EHI_126560_at | 3.1 | Upregulated | 1101698136470 13765 bp 1 |
| EHI_176700_at | 4.1 | Downregulated | 1101698136491 9654 bp 2 c |
| EHI_176580_at | 3.5 | Downregulated | 1101698136491 9654 bp 2 c |
| EHI_136840_s_at | 7.3 | Upregulated | 1101698136528 1275 bp 1 c |
| EHI_127670_at | 17 | Downregulated | 1101698136656 13077 bp 2 |
| EHI_059280_s_at | 6 | upregulated | 1101698136833 20101 bp 1 |
| EHI_087210_at | 8.9 | upregulated | 1101698136862 4399 bp 1 c |
| EHI_018270_s_at | 5.9 | upregulated | 1101698136874 10045 bp 3 |
| EHI_100250_at | 4.2 | upregulated | 1101698136880 74468 bp 3 |
| EHI_037700_s_at | 6 | upregulated | 1101698136881 9481 bp 1 c |
| EHI_034530_s_at | 5.1 | upregulated | 1101698136891 23360 bp 1 |
| EHI_034590_s_at | 4.6 | upregulated | 1101698136891 23360 bp 1 |
| EHI_091450_at | 3 | upregulated | 1101698136921 22165 bp 1 |
| EHI_074750_at | 3.9 | upregulated | 1101698136931 22083 bp 1 |
| EHI_026000_s_at | 6.5 | upregulated | 1101698136940 11576 bp 1 |
| EHI_167450_s_at | 4.1 | upregulated | 1101698136967 11956 bp 1 |
| EHI_109250_s_at | 5.9 | downregulated | 1101698136991 32769 bp 1 |
| EHI_062960_at | 4 | downregulated | 1101698136993 27728 bp 1 |
| EHI_069940_at | 3.3 | downregulated | 1101698136998 17014 bp 1 |
| EHI_025710_at | 11.6 | upregulated | 1101698137001 11966 bp 1 |
| EHI_141050_at | 4.7 | upregulated | 1101698137002 47053 bp 1 |
| EHI_141030_at | 4.1 | upregulated | 1101698137002 47053 bp 1 |
| EHI_067250_at | 6.7 | downregulated | 1101698137005 16852 bp 1 |
| EHI_067260_at | 4.3 | downregulated | 1101698137005 16852 bp 1 |
| EHI_067220_at | 3 | downregulated | 1101698137005 16852 bp 1 |
| EHI_090260_at | 3.4 | downregulated | 1101698137010 27630 bp 2 |
| EHI_067720_s_at | 4.8 | upregulated | 1101698137059 20106 bp 1 |
| EHI_191730_at | 5.4 | upregulated | 1101698137083 15331 bp 2 |
| EHI_075710_at | 3.9 | upregulated | 1101698137107 22938 bp 1 |
| EHI_075660_at | 3.6 | upregulated | 1101698137107 22938 bp 1 |
| EHI_075640_at | 3.1 | upregulated | 1101698137107 22938 bp 1 |
| EHI_160330_s_at | 7.3 | downregulated | 1101698137110 20771 bp 2 |
| EHI_047630_s_at | 5.7 | downregulated | 1101698137137 3916 bp 1 c |
| EHI_114950_at | 4.2 | downregulated | 1101698137177 31689 bp 3 |
| EHI_114650_at | 3.3 | upregulated | 1101698137177 31689 bp 3 |
| EHI_006850_at | 36.9 | upregulated | 1101698137185 77296 bp 2 |
| EHI_006140_at | 3.2 | downregulated | 1101698137191 58118 bp 1 |
| EHI_074520_s_at | 5.5 | upregulated | 1101698137214 22866 bp 1 |
| EHI_020250_at | 3.3 | downregulated | 1101698137226 31684 bp 1 |
| EHI_096770_at | 4.9 | upregulated | 1101698137228 132287 bp 3 |
| EHI_189960_at | 7.2 | upregulated | 1101698137229 35581 bp 1 |
| EHI_148550_at | 3.8 | upregulated | 1101698137233 210398 bp 2 |
| EHI_012990_at | 3.2 | upregulated | 1101698137237 118395 bp 6 |
| EHI_026360_s_at | 3.2 | downregulated | 1101698137239 42641 bp 3 |
| EHI_129830_at | 6.2 | downregulated | 1101698137242 43091 bp 1 |
| EHI_129890_at | 5.4 | upregulated | 1101698137242 43091 bp 1 |
| EHI_129880_at | 3.1 | upregulated | 1101698137242 43091 bp 1 |
| EHI_075150_at | 3.6 | upregulated | 1101698137249 16901 bp 2 |
| EHI_156680_at | 3.2 | upregulated | 1101698137250 11527 bp 2 |
| EHI_138480_at | 6.3 | upregulated | 1101698137256 53356 bp 2 |
| EHI_050490_at | 9.1 | upregulated | 1101698137258 185630 bp 3 |
| EHI_073980_s_at | 6.2 | upregulated | 1101698137262 56534 bp 2 |
| EHI_180390_at | 5 | downregulated | 1101698137264 61582 bp 3 |
| EHI_162780_s_at | 6 | upregulated | 1101698137266 7809 bp 1 c |
| EHI_137240_at | 3.4 | downregulated | 1101698137276 42629 bp 4 |
| EHI_022270_s_at | 5.5 | upregulated | 1101698137297 21066 bp 2 |
| EHI_054690_at | 5.8 | downregulated | 1101698137320 58471 bp 5 |
| EHI_054680_at | 4.8 | downregulated | 1101698137320 58471 bp 5 |
| EHI_054700_at | 3.3 | downregulated | 1101698137320 58471 bp 5 |
| EHI_174600_at | 3.2 | upregulated | 1101698137321 84158 bp 5 |
| EHI_196720_s_at | 10.9 | upregulated | 1101698137324 14794 bp 1 |
| EHI_002240_s_at | 11.4 | upregulated | 1101698137326 31028 bp 1 |
| EHI_072000_s_at | 4.8 | upregulated | 1101698137336 41777 bp 1 |
| EHI_032670_s_at | 3.3 | upregulated | 1101698137341 36157 bp 6 |
| EHI_077280_s_at | 163.1 | downregulated | 1101698137342 57228 bp 5 |
| EHI_004520_at | 3.8 | upregulated | 1101698137345 93434 bp 6 |
| EHI_152200_at | 3.2 | downregulated | 1101698137352 530629 bp 4 |
| EHI_091350_s_at | 5.9 | upregulated | 1101698137359 3635 bp 1 c |
| EHI_165190_at | 27.1 | upregulated | 1101698137361 62837 bp 3 |
| EHI_165200_at | 4 | upregulated | 1101698137361 62837 bp 3 |
| EHI_118420_at | 9.5 | upregulated | 1101698137366 94149 bp 2 |
| EHI_118410_at | 3.1 | upregulated | 1101698137366 94149 bp 2 |
| EHI_095480_at | 3.2 | upregulated | 1101698137375 20664 bp 2 |
| EHI_067600_at | 3.1 | downregulated | 1101698137377 21871 bp 2 |
| EHI_045450_at | 3.1 | upregulated | 1101698137390 188109 bp 5 |
| EHI_045600_at | 3.1 | upregulated | 1101698137390 188109 bp 5 |
| EHI_179060_at | 3.6 | downregulated | 1101698137407 159841 bp 3 |
| EHI_010130_at | 4.4 | upregulated | 1101698137427 74661 bp 2 |
| EHI_147860_at | 7.2 | downregulated | 1101698137428 24646 bp 2 |
| EHI_082060_at | 3.4 | upregulated | 1101698137430 42861 bp 5 |
| EHI_029500_s_at | 6.8 | upregulated | 1101698137440 31365 bp 1 |
| EHI_029620_s_at | 3.1 | upregulated | 1101698137440 31365 bp 1 |
| EHI_187080_at | 5.1 | downregulated | 1101698137444 145071 bp 4 |
| EHI_073520_at | 3.7 | downregulated | 1101698137448 109509 bp 3 |
| EHI_159810_s_at | 11.7 | downregulated | 1101698137449 120372 bp 9 |
| EHI_022600_s_at | 5.4 | upregulated | 1101698137450 163464 bp 3 |
| EHI_023150_at | 3 | upregulated | 1101698137450 163464 bp 3 |
| EHI_011560_s_at | 6.7 | upregulated | 1101698137454 21257 bp 1 |
| EHI_164190_at | 12.9 | upregulated | 1101698137460 65518 bp 5 |
| EHI_164170_s_at | 4.9 | upregulated | 1101698137460 65518 bp 5 |
| EHI_174230_s_at | 4.1 | downregulated | 1101698137462 61702 bp 6 |
| EHI_049960_at | 3 | downregulated | 1101698137474 165291 bp 5 |
| EHI_033560_s_at | 171.4 | downregulated | 1101698137483 49838 bp 3 |
| EHI_183210_s_at | 9.1 | upregulated | 1101698137489 154425 bp 3 |
| EHI_058480_at | 3.3 | downregulated | 1101698137490 44332 bp 5 |
| EHI_092100_at | 3.2 | upregulated | 1101698137492 156102 bp 7 |
| EHI_166690_at | 6.5 | upregulated | 1101698137494 95967 bp 5 |
| EHI_103260_s_at | 4.5 | upregulated | 1101698137495 145104 bp 3 |
