# Supplementary figures and images for: Phenotypic and transcriptional profiling in Entamoeba histolytica reveal costs to fitness and adaptive responses associated with metronidazole resistance
Source: Front Microbiol. 2015 May 5;6:354. doi: 10.3389/fmicb.2015.00354 (PMC4419850; doi:10.3389/fmicb.2015.00354)

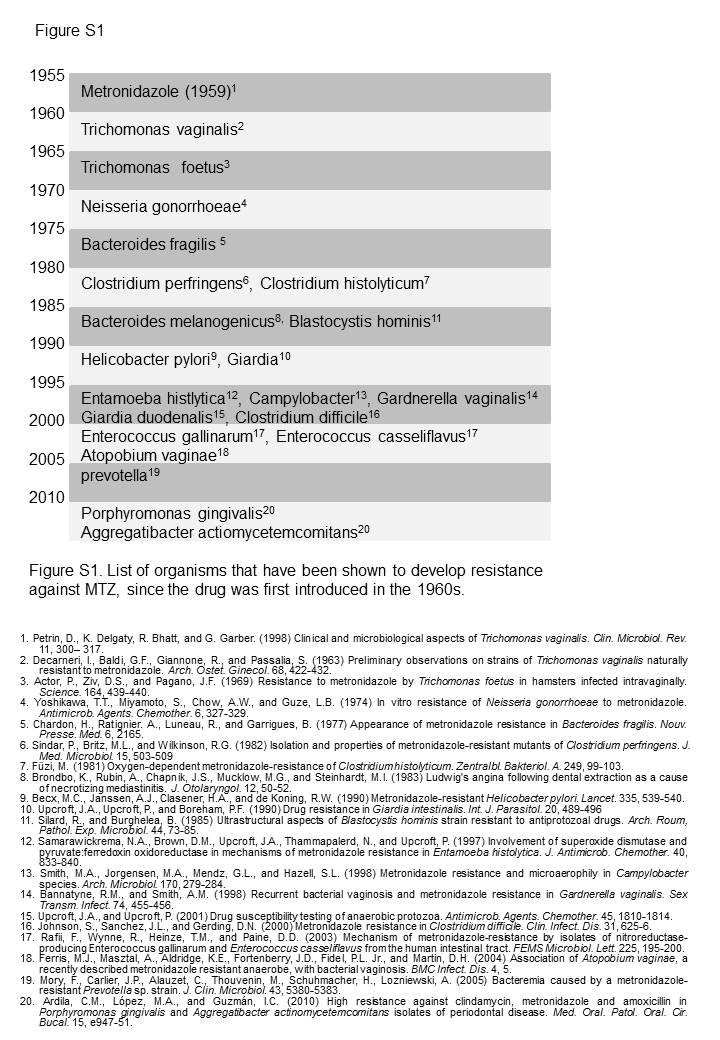

Supplement: Supplementary file 8 [file Image1.JPEG]

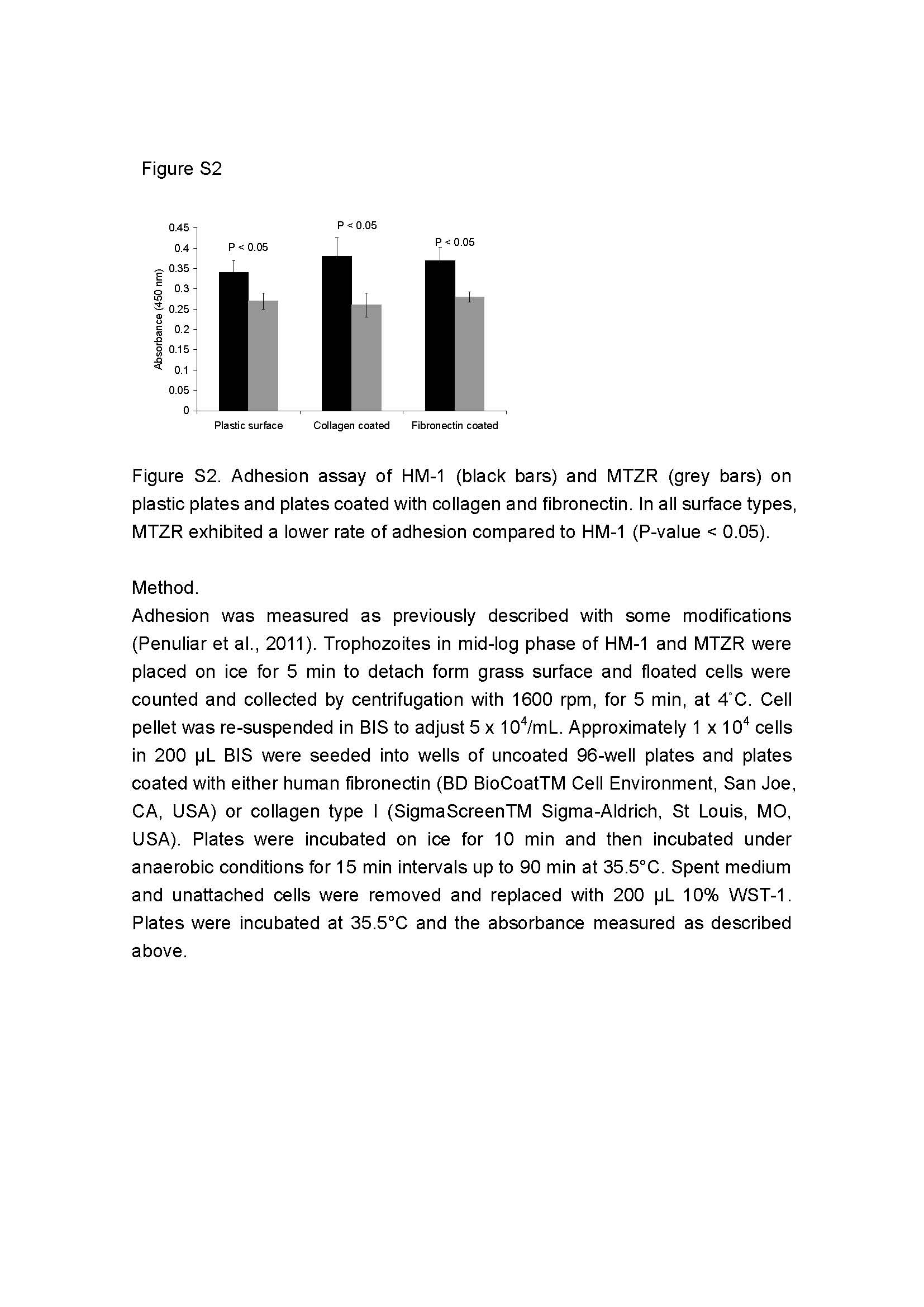

Supplement: Supplementary file 9 [file Image2.JPEG]
